# Supplementary material for: Sleep cycle-dependent vascular dynamics in male mice and the predicted effects on perivascular cerebrospinal fluid flow and solute transport
Source: Nat Commun. 2023 Feb 20;14:953. doi: 10.1038/s41467-023-36643-5 (PMC9941497; doi:10.1038/s41467-023-36643-5)
Supplement: Supplementary file 1 — Supplementary Information [file 41467_2023_36643_MOESM1_ESM.pdf]

Sleep cycle-dependent vascular dynamics in male mice and the predicted effects on perivascular cerebrospinal fluid flow and solute transport

Laura Bojarskaite, Alexandra Vallet, Daniel M. Bjørnstad, Kristin M. Gullestad Binder, Céline Cunen, Kjell Heuser, Miroslav Kuchta, Kent-Andre Mardal, Rune Enger

Supplementary Information

This supplementary information contains:

Supplementary figures 1–19
Supplementary tables 1–10
Statistical test details: effect sizes, degrees of freedom and p-values

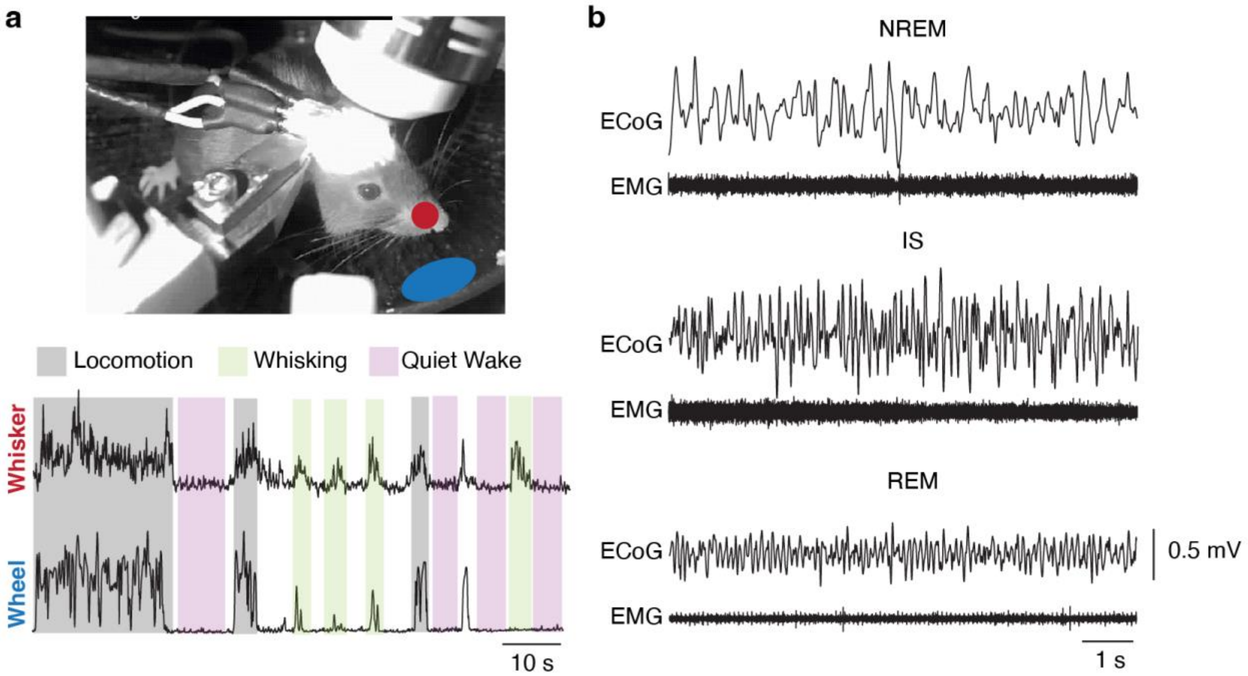

Supplementary Fig. 1: Sleep-wake state scoring. a, Wakefulness was separated into locomotion, whisking and quiet wakefulness based on movement detected in snout and running wheel regions in the surveillance video. **b,** Representative ECoG and EMG traces during NREM, IS and REM sleep. NREM: non-rapid eye movement sleep; IS: intermediate state sleep; REM: rapid eye movement sleep; ECoG: electrocorticography; EMG: electromyography.

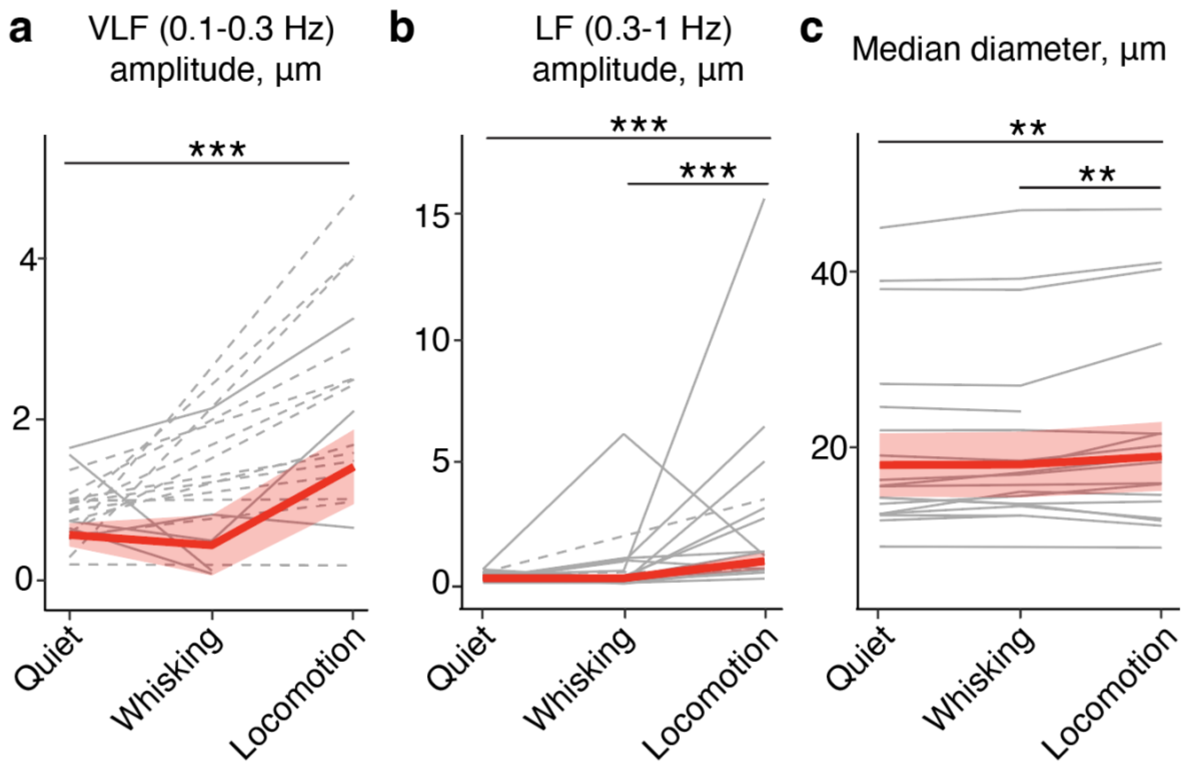

Supplementary Fig. 2: a, Amplitude of very low frequency (VLF 0.1–0.3 Hz) oscillations, **b,** amplitude of low frequency (LF 0.3-1 Hz) oscillations, and **c,** median diameter of lumen of pial arteries during quiet wakefulness, whisking and locomotion. Every gray line (dashed or full) represents an individual vessel, dashed lines are used when an observation is missing in a certain state for a given vessel (for example dashed line between Quiet and Locomotion means that we do not have an observation for that particular vessel in Whisking state), bold lines and shaded area are the estimates and 95% CI from linear mixed effects models (two-sided). For **a**: n = 498 episodes, 19 vessels, 5 mice; for **b**: n = 797 episodes, 19 vessels, 5 mice; for **c** n = 888 episodes, 19 vessels, 5 mice. $*P < 0.05$, $**P < 0.01$, $***P < 0.001$, Tukey adjustment for multiple comparisons. See end of supplementary information for exact p-values, and degrees of freedom for the statistical tests. Source data are provided as a Source Data file.

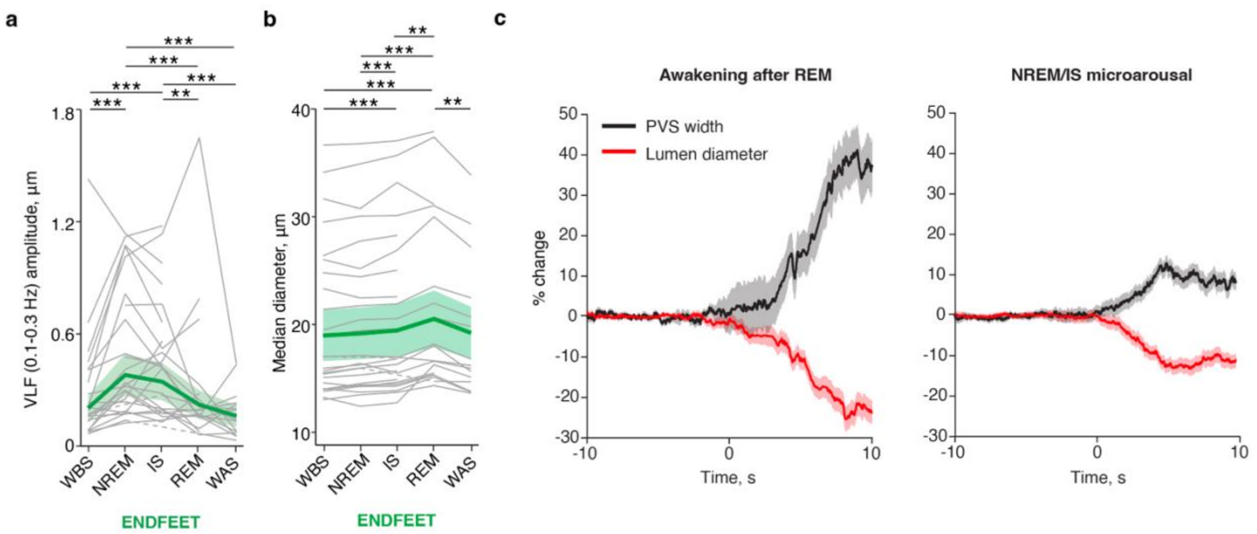

Supplementary Fig. 3: **a,** Amplitude of very low frequency (VLF 0.1–0.3 Hz) oscillations and **b,** median diameter of enfoot tube during a sleep cycle. Every gray line (dashed or full) represents an individual vessel, dashed lines are used when an observation is missing in a certain state for a given vessel (for example dashed line between WBS and IS means that we do not have an observation for that particular vessel in NREM state), bold lines and shaded area are the estimates and 95% CI from linear mixed effects models (two-sided). **c,** Median percentage change in PVS width and lumen diameter of penetrating arterioles during brief awakening from REM sleep (*left*) and during microarousals in NREM and IS sleep (*right*). For **a**: n = 306 episodes, 25 vessels, 4 mice; for **b**: n = 310 episodes, 25 vessels, 4 mice; for **c**: n = 5 mice, 15 awakenings after REM, 90 microarousals. $*P < 0.05$, $**P < 0.01$, $***P < 0.001$, Tukey adjustment for multiple comparisons. WBS: wake before sleep; NREM: non-rapid eye movement sleep; IS: intermediate state sleep; REM: rapid eye movement sleep; WAS: wake after sleep; PVS: perivascular space. See end of supplementary information for exact p-values, and degrees of freedom for the statistical tests. Source data are provided as a Source Data file.

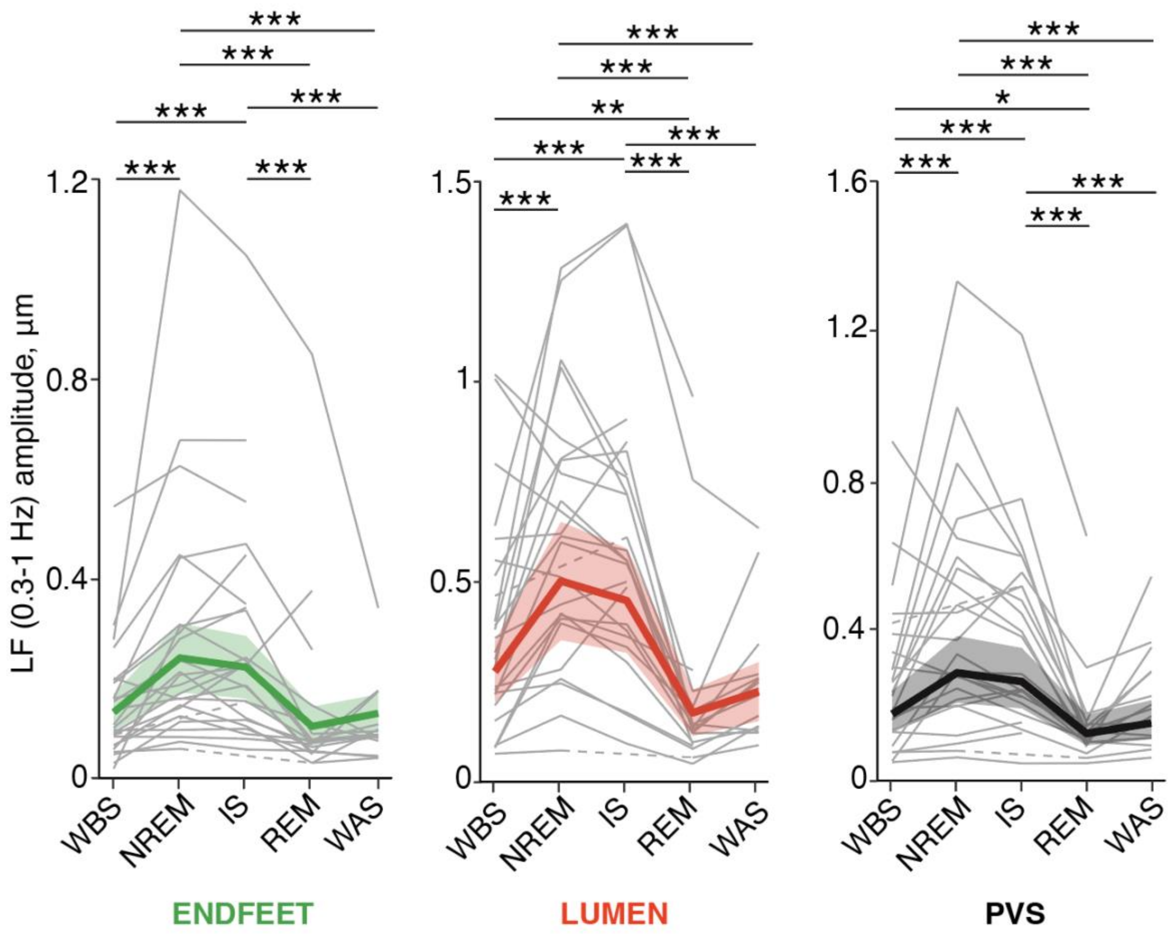

Supplementary Fig. 4: Amplitude of low frequency (LF 0.3–1 Hz) oscillations in endfoot tube, lumen and PVS during a sleep cycle. Every gray line (dashed or full) represents an individual vessel, dashed lines are used when an observation is missing in a certain state for a given vessel (for example dashed line between WBS and IS means that we do not have an observation for that particular vessel in NREM state), bold lines and shaded area are the estimates and 95% CI from linear mixed effects models (two-sided), n = 310 episodes, 25 vessels, 4 mice. $*P < 0.05$, $**P < 0.01$, $***P < 0.001$, Tukey adjustment for multiple comparisons. WBS: wake before sleep; NREM: non-rapid eye movement sleep; IS: intermediate state sleep; REM: rapid eye movement sleep; WAS: wake after sleep; PVS: perivascular space. See end of supplementary information for exact p-values, and degrees of freedom for the statistical tests. Source data are provided as a Source Data file.

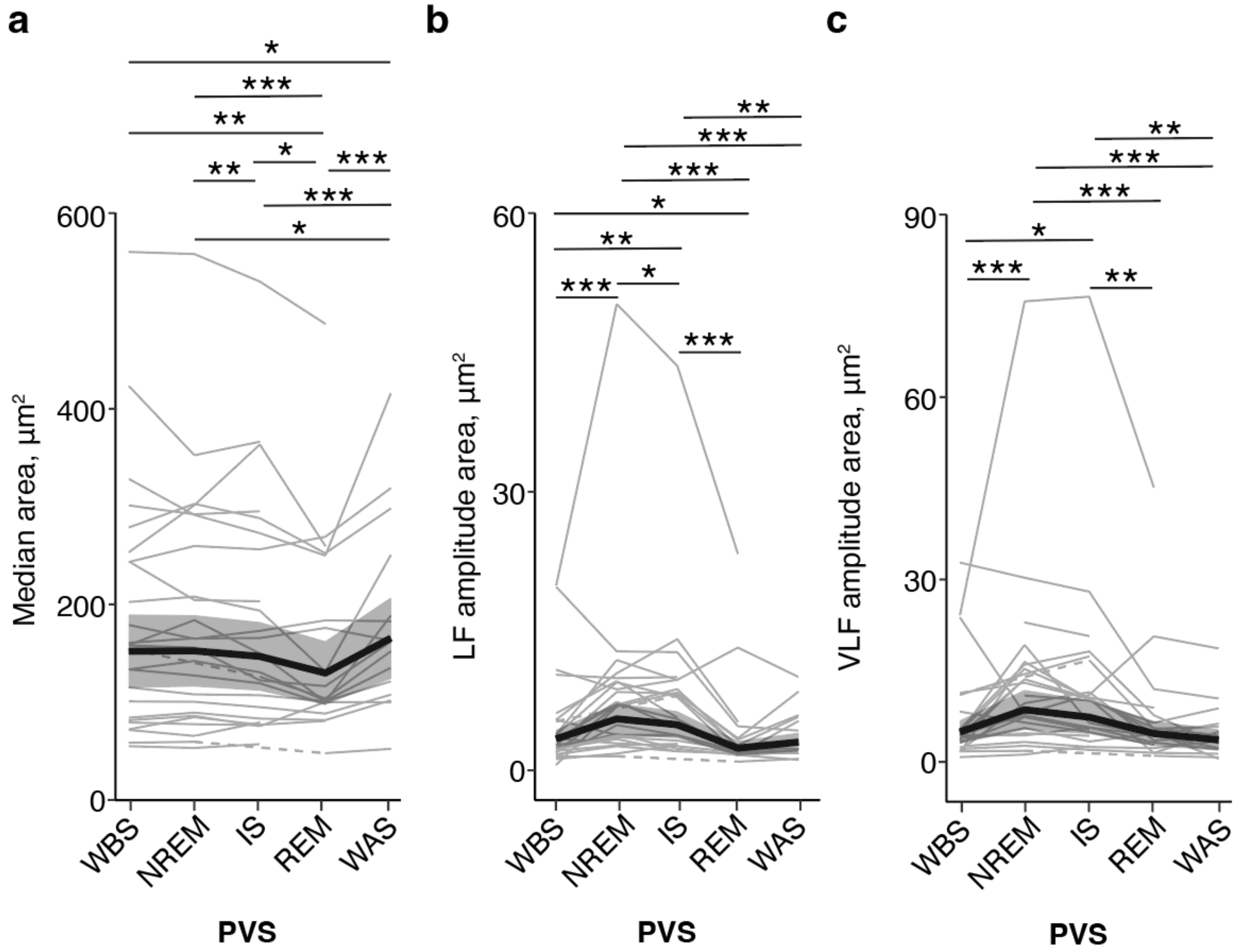

Supplementary Fig. 5: **a,** Median PVS area, **b,** LF amplitude in PVS area and **c,** VLF amplitude in PVS area during a sleep cycle. Every gray line (dashed or full) represents an individual vessel, dashed lines are used when an observation is missing in a certain state for a given vessel (for example dashed line between WBS and IS means that we do not have an observation for that particular vessel in NREM state), bold lines and shaded area are the estimates and 95% CI from linear mixed effects models (two-sided). For **a** and **b**: n = 310 episodes, 25 vessels, 4 mice; for **c**: n = 304 episodes, 25 vessels, 4 mice. *$P < 0.05$, **$P < 0.01$, ***$P < 0.001$, Tukey adjustment for multiple comparisons. WBS: wake before sleep; NREM: non-rapid eye movement sleep; IS: intermediate state sleep; REM: rapid eye movement sleep; WAS: wake after sleep; LF: low frequency; PVS: perivascular space. See end of supplementary information for exact p-values, and degrees of freedom for the statistical tests. Source data are provided as a Source Data file.

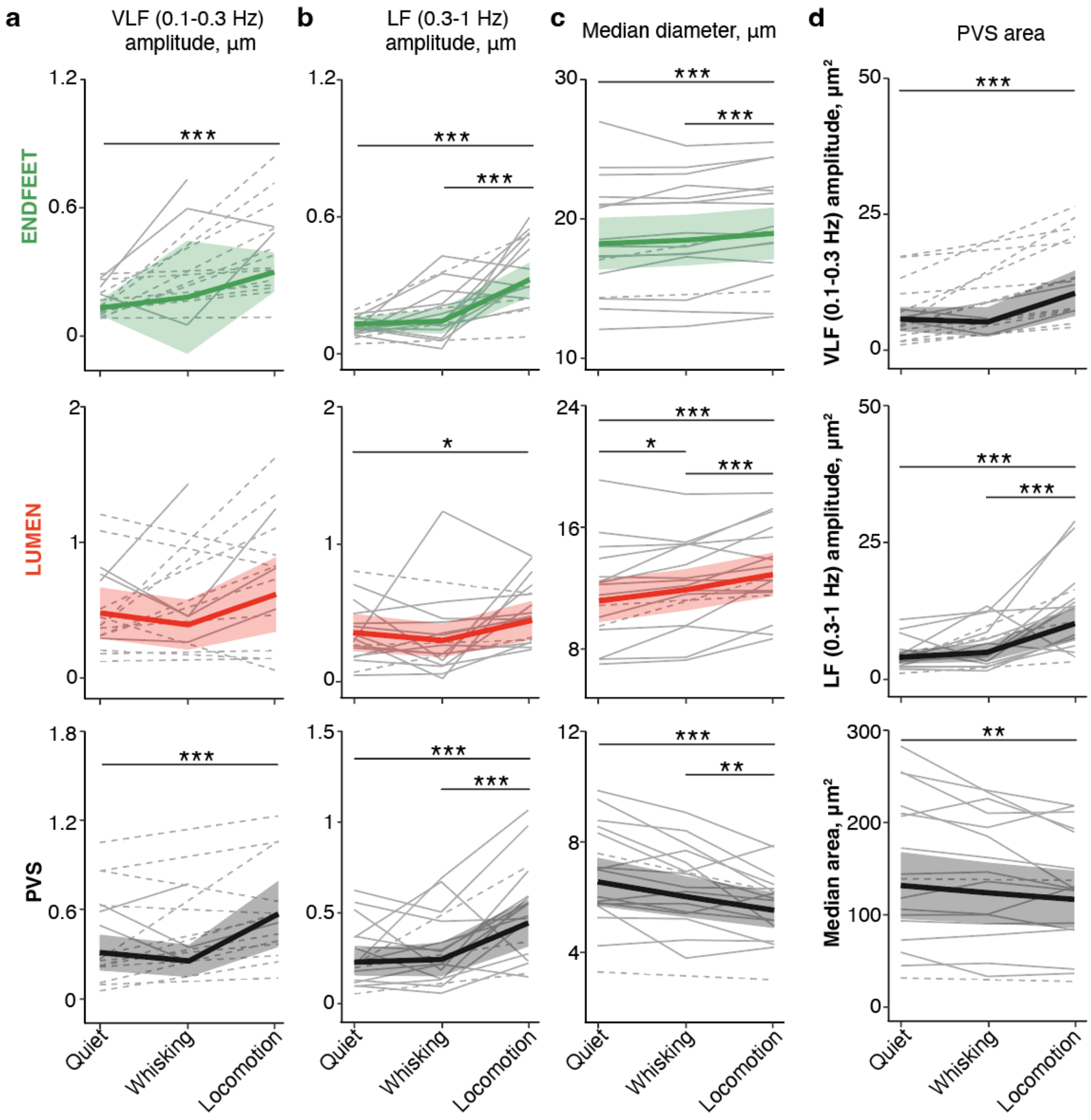

Supplementary Fig. 6: a, Amplitude of very low frequency (VLF 0.1–0.3 Hz) oscillations, **b,** amplitude of low frequency (LF 0.3–1 Hz) oscillations, and **c,** median diameter (width for PVS) of (*top-to-bottom*) endfoot tube, lumen and PVS of penetrating arterioles during quiet wakefulness, whisking and locomotion. **d,** (*top-to-bottom*) VLF amplitude in PVS area, LF amplitude in PVS area and median PVS area during quiet wakefulness, whisking and locomotion. Every gray line (dashed or full) represents an individual vessel, dashed lines are used when an observation is missing in a certain state for a given vessel (for example dashed line between Quiet and

Locomotion means that we do not have an observation for that particular vessel in Whisking state), bold lines and shaded area are the estimates and 95% CI from linear mixed effects models (two-sided). **a**: n = 127 episodes, 16 vessels, 3 mice for endfoot tube and lumen, n = 134 episodes, 16 vessels, 3 mice for PVS; **b:** n = 236 episodes, 17 vessels, 3 mice for endfoot tube, n = 228 episodes, 17 vessels, 3 mice for lumen, n = 244 episodes, 17 vessels, 3 mice for PVS; **c**: n = 262 episodes, 17 vessels, 3 mice **d**: n = 134 episodes, 16 vessels, 3 mice for VLF amplitude, n = 243 episodes, 17 vessels, 3 mice for LF amplitude, n = 262 episodes, 17 vessels, 3 mice for median area. *$P < 0.05$, **$P < 0.01$, ***$P < 0.001$, Tukey adjustment for multiple comparisons. See end of supplementary information for exact p-values, and degrees of freedom for the statistical tests. PVS: perivascular space. Source data are provided as a Source Data file.

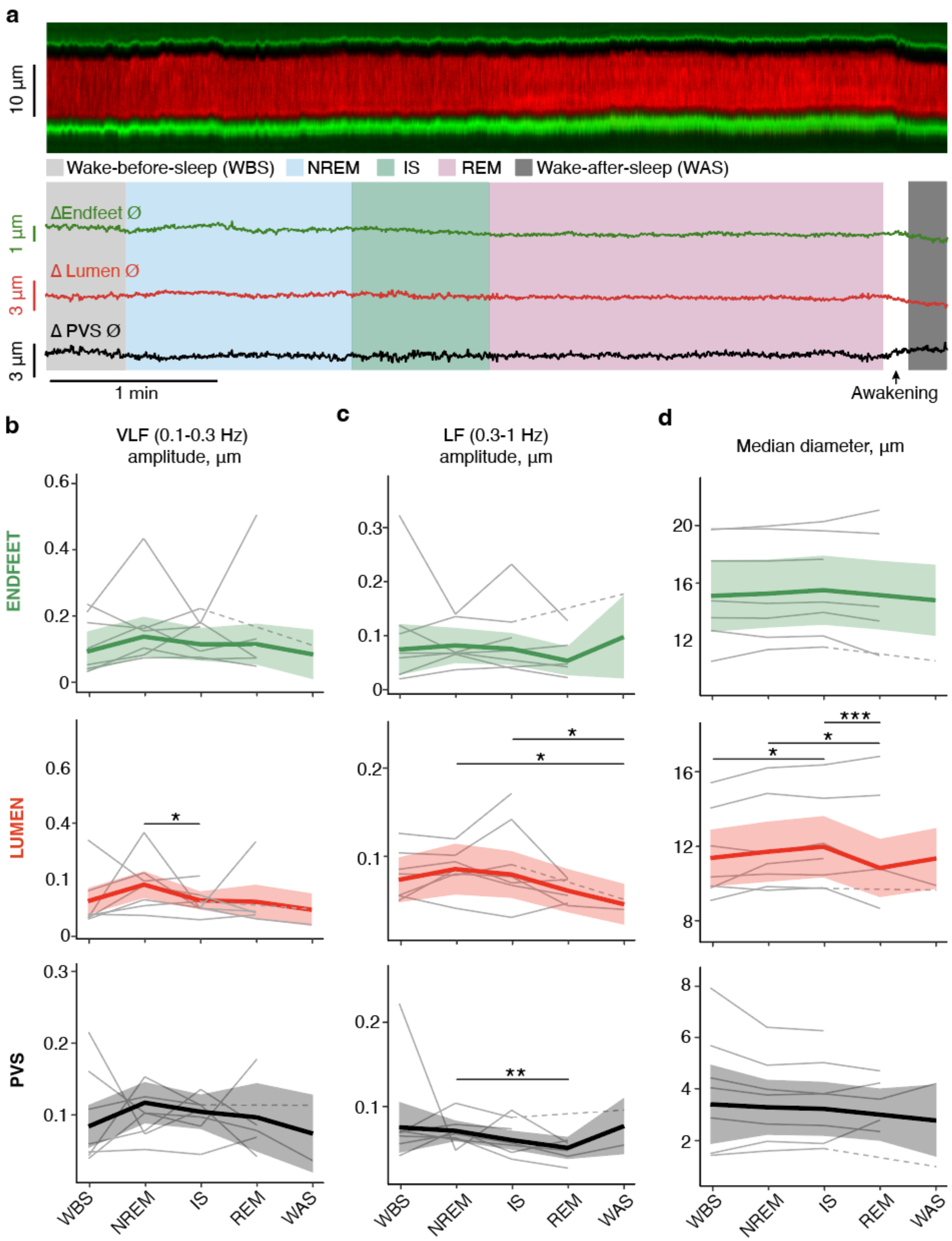

Supplementary Fig. 7: a, Representative traces of a venule line scan, endfoot tube diameter, vessel lumen diameter and PVS width during a sleep cycle. **b,** Amplitude of very low frequency

(VLF 0.1–0.3 Hz) oscillations, **c,** amplitude of low frequency (LF 0.3–1 Hz) oscillations, and **d,** median diameter (width for PVS) of (*top-to-bottom*) endfoot tube, lumen and PVS of venules throughout a sleep cycle. Every gray line (dashed or full) represents an individual vessel, dashed lines are used when an observation is missing in a certain state for a given vessel (for example dashed line between WBS and IS means that we do not have an observation for that particular vessel in NREM state), bold lines and shaded area are the estimates and 95% CI from linear mixed effects models (two-sided), n = 90 episodes, 7 vessels, 3 mice. *$P < 0.05$, **$P < 0.01$, ***$P < 0.001$, Tukey adjustment for multiple comparisons. WBS: wake before sleep; NREM: non-rapid eye movement sleep; IS: intermediate state sleep; REM: rapid eye movement sleep; WAS: wake after sleep; PVS: perivascular space. See end of supplementary information for exact p-values, and degrees of freedom for the statistical tests. Source data are provided as a Source Data file.

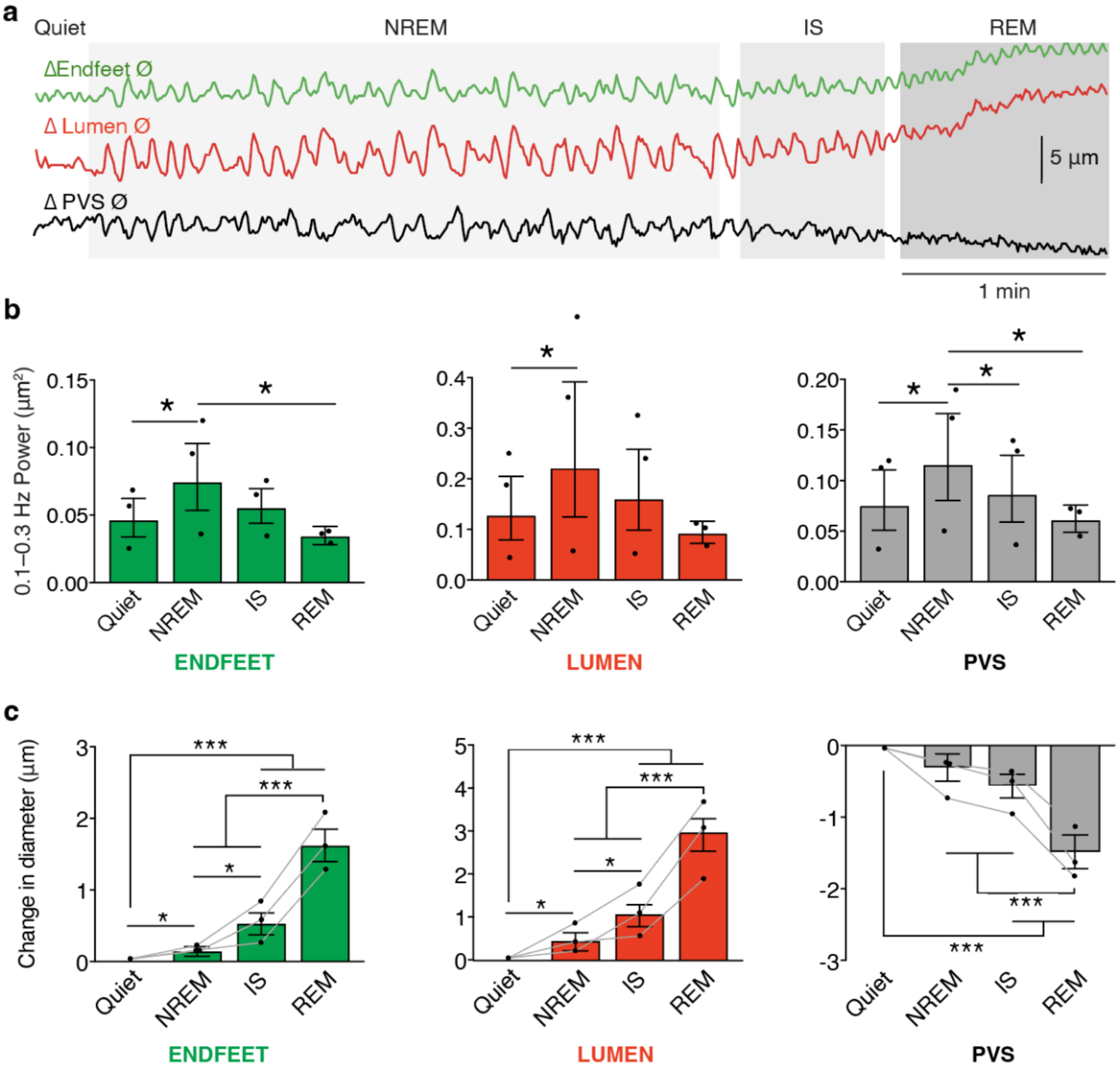

Supplementary Fig. 8: 2D time-series analysis of vascular dynamics across the sleep cycle. a, Representative traces of a penetrating arteriole endfoot tube diameter, vessel lumen diameter and PVS total width during quiet wakefulness, NREM, IS and REM sleep states. **b,** Diameter oscillation power in the 0.1–0.3 Hz frequency range of endfoot tube, vessel lumen and PVS during quiet wakefulness, NREM, IS and REM sleep. Data represented as estimates ± standard error, n = 3 mice, 72 arterioles. Estimates from each mouse is scattered on top of the bar graphs. **c,** Change in the median diameter per sleep state of the endfoot tube, vessel lumen, and perivascular space of penetrating arterioles from quiet wakefulness to NREM, IS and REM sleep. Data represented as

estimates from linear mixed effects model (two-sided, no adjustment for multiple comparisons) ± SE, n = 3 mice, 136 arterioles. *$P < 0.05$, **$P < 0.01$, ***$P < 0.001$. NREM: non-rapid eye movement sleep; IS: intermediate state sleep; REM: rapid eye movement sleep; PVS: perivascular space. Source data are provided as a Source Data file.

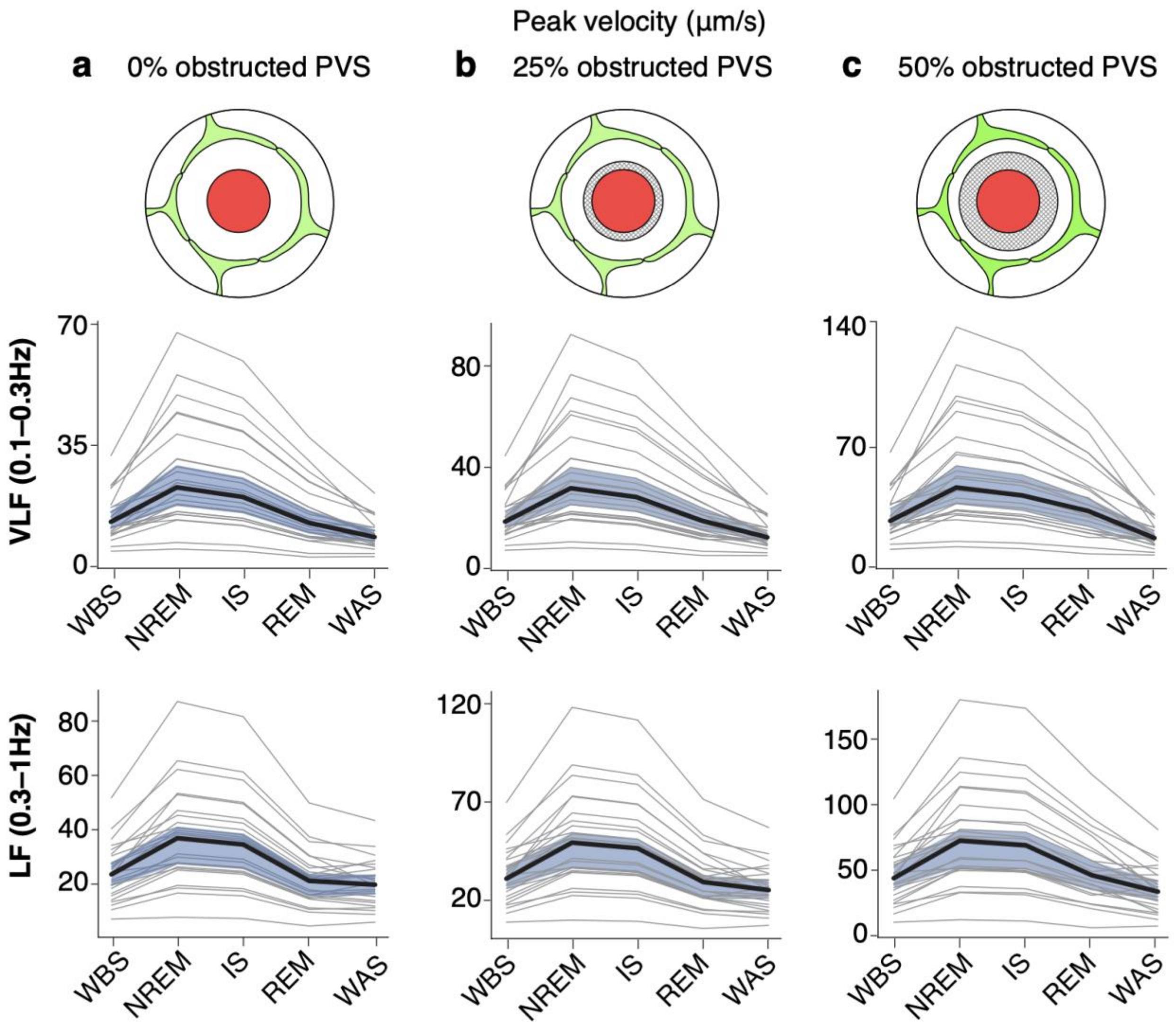

Supplementary Fig. 9: NREM/IS slow vasomotion increases predicted PVS fluid velocity. a-c, Peak fluid velocity in penetrating arteriole PVS generated by VLF and LF oscillations during a sleep cycle as predicted by biomechanical modeling using three where we added a fixed volume to the PVS obstructing flow corresponding to 0%, 25% and 50% of our measured PVS in quiet wakefulness. Gray lines represent observations from individual penetrating arterioles while bolded black lines with the blue shading are median values with 10th and 90th percentiles, n = 16 vessels, 4 mice. WBS: wake before sleep; NREM: non-rapid eye movement sleep; IS: intermediate state sleep; REM: rapid eye movement sleep; WAS: wake after sleep; PVS: perivascular space; VLF: very low frequency; LF: low frequency. Source data are provided as a Source Data file.

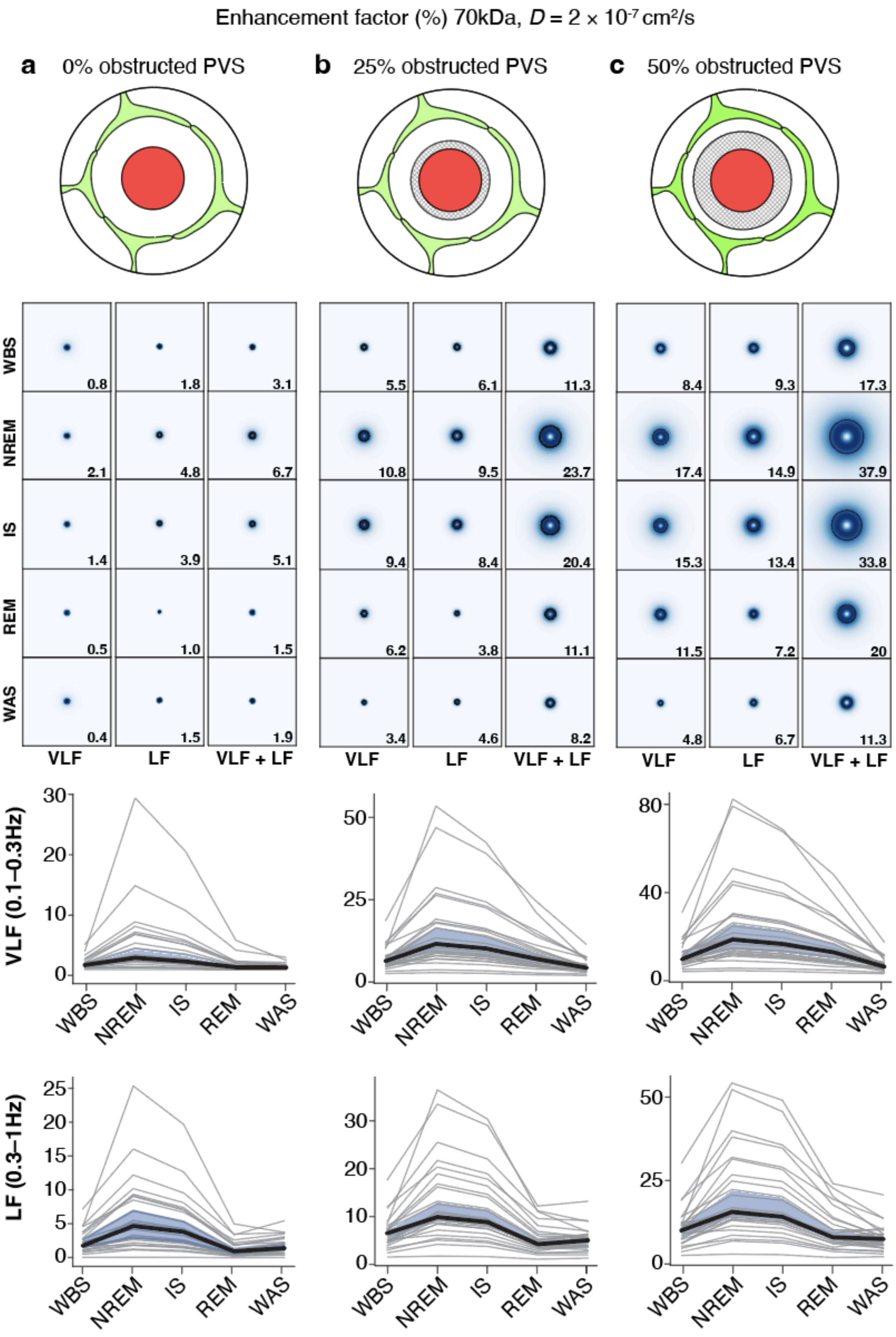

Enhancement factor (%) 70kDa, D = 2 × 10-7 cm²/s
a 0% obstructed PVS
b 25% obstructed PVS
c 50% obstructed PVS
WBS
NREM
IS
REM
WAS
0.8
1.8
3.1
2.1
4.8
6.7
1.4
3.9
5.1
0.5
1.0
1.5
0.4
1.5
1.9
5.5
6.1
11.3
10.8
9.5
23.7
9.4
8.4
20.4
6.2
3.8
11.1
3.4
4.6
8.2
8.4
9.3
17.3
17.4
14.9
37.9
15.3
13.4
33.8
11.5
7.2
20
4.8
6.7
11.3
VLF
LF
VLF + LF
VLF (0.1–0.3Hz)
LF (0.3–1Hz)

Supplementary Fig. 10. NREM/IS slow vasomotion enhances predicted dispersion in PVS. a-c, Enhancement factor for 70 kDa tracer spread in penetrating arteriole PVS generated by VLF and LF oscillations during a sleep cycle as predicted by biomechanical modeling using three scenarios where we added a fixed volume to the PVS obstructing flow corresponding to 0%, 25% and 50% of our measured PVS in quiet wakefulness. The enhancement factor is the relative increase in solute movement induced by VLF and LF oscillatory flow, compared to pure diffusion. The black circle represents the median, whereas the shading represents the distribution of all modeled vessels (n = 16 vessels, 4 mice). Gray lines represent observations from individual penetrating arterioles while bolded black lines with the blue shading are median values with 10th and 90th percentiles, n = 16 vessels, 4 mice. WBS: wake before sleep; NREM: non-rapid eye movement sleep; IS: intermediate state sleep; REM: rapid eye movement sleep; WAS: wake after sleep; PVS: perivascular space; VLF: very low frequency; LF: low frequency. Source data are provided as a Source Data file.

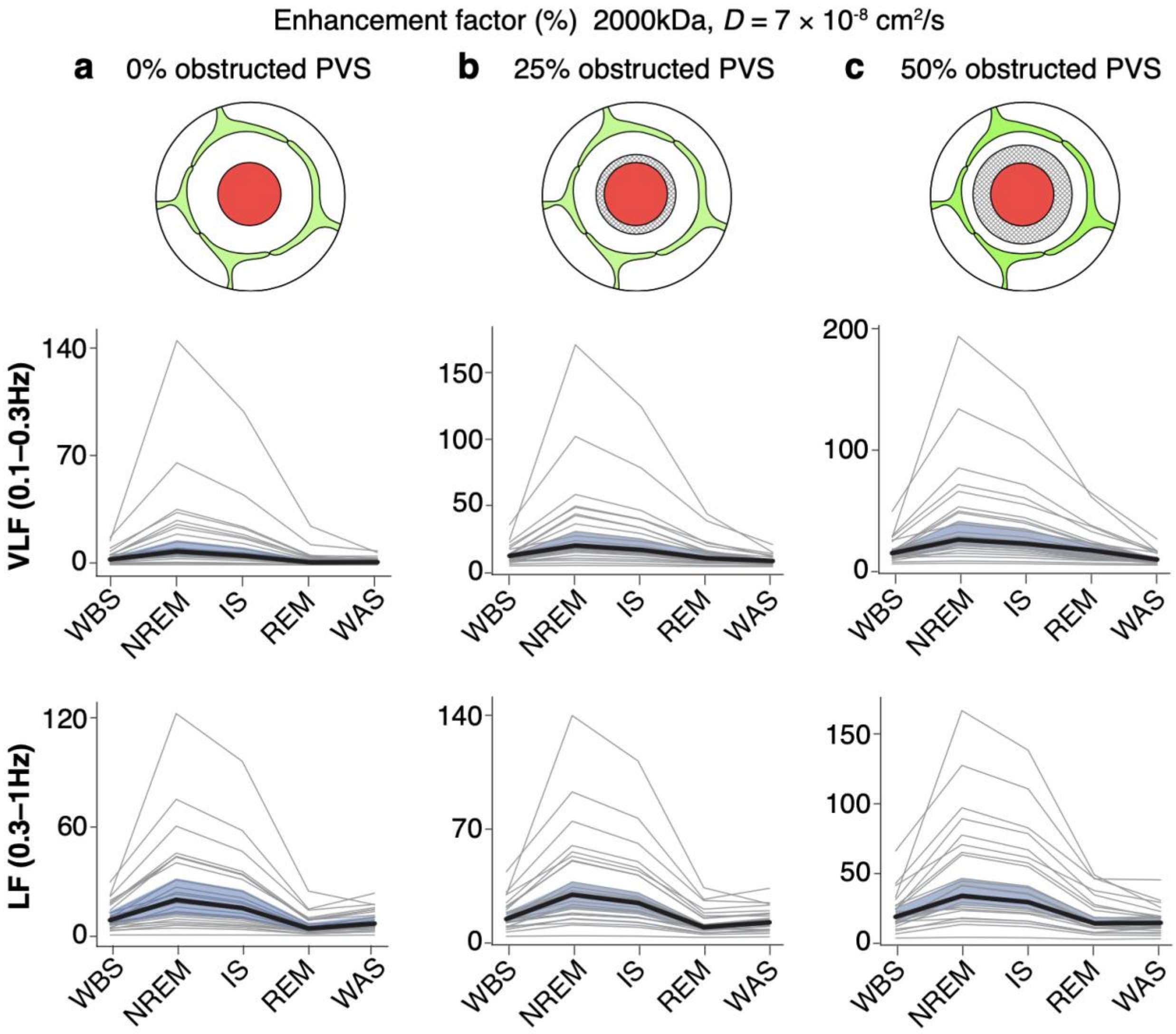

Supplementary Fig. 11: **NREM/IS slow vasomotion enhances predicted dispersion in PVS. a-c,** Enhancement factor for 2000 kDa tracer spread in penetrating arteriole PVS generated by VLF and LF oscillations during a sleep cycle as predicted by biomechanical modeling using three scenarios where we added a fixed volume to the PVS obstructing flow corresponding to 0%, 25% and 50% of our measured PVS in quiet wakefulness. The enhancement factor is the relative increase in solute movement induced by VLF and LF oscillatory flow, compared to pure diffusion. Gray lines represent observations from individual penetrating arterioles while bolded black lines with the blue shading are median values with 10th and 90th percentiles, n = 16 vessels, 4 mice. WBS: wake before sleep; NREM: non-rapid eye movement sleep; IS: intermediate state sleep; REM: rapid eye movement sleep; WAS: wake after sleep; PVS: perivascular space; VLF: very low frequency; LF: low frequency. Source data are provided as a Source Data file.

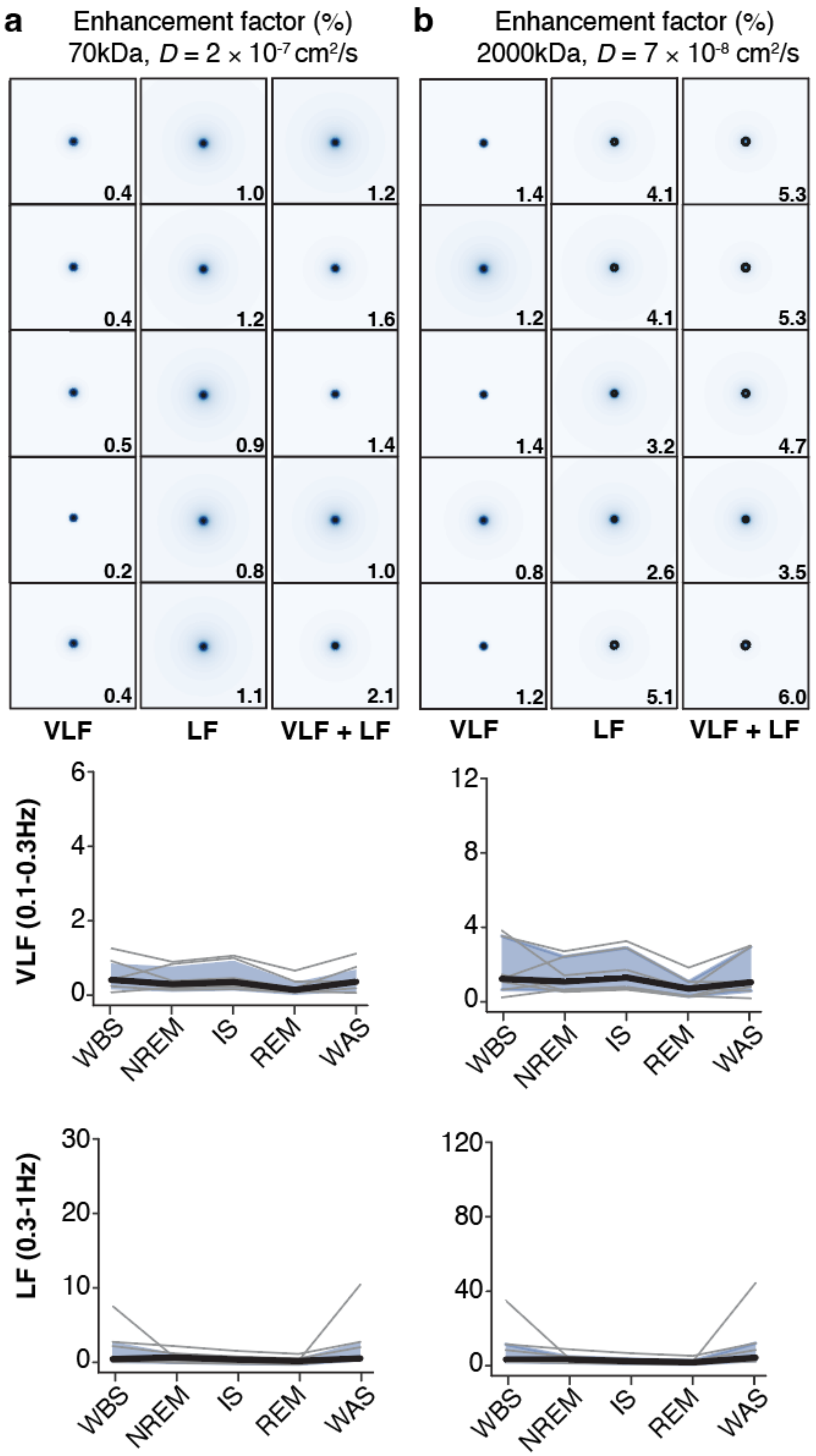

a
Enhancement factor (%)
70kDa, D = 2 × 10-7 cm2/s
b
Enhancement factor (%)
2000kDa, D = 7 × 10-8 cm2/s
0.4
1.0
1.2
1.4
4.1
5.3
0.4
1.2
1.6
1.2
4.1
5.3
0.5
0.9
1.4
1.4
3.2
4.7
0.2
0.8
1.0
0.8
2.6
3.5
0.4
1.1
2.1
1.2
5.1
6.0
VLF
LF
VLF + LF
VLF
LF
VLF + LF
VLF (0.1-0.3Hz)
LF (0.3-1Hz)
WBS
NREM
IS
REM
WAS

Supplementary Fig. 12: **Predicted dispersion of solutes across sleep-wake cycle in venule PVS. a-b,** Enhancement factor for 70 kDa and 2000 kDa tracer spread in venule PVS generated by VLF and LF oscillations during a sleep cycle as predicted by biomechanical modeling. The black circle represents the median, whereas the shading represents the distribution of all modeled vessels. The enhancement factor is the relative increase in solute movement induced by VLF and LF oscillatory flow, compared to pure diffusion. Gray lines represent observations from individual penetrating arterioles while bolded black lines with the blue shading are median values with 10th and 90th percentiles, n = 7 vessels, 4 mice. WBS: wake before sleep; NREM: non-rapid eye movement sleep; IS: intermediate state sleep; REM: rapid eye movement sleep; WAS: wake after sleep; PVS: perivascular space; VLF: very low frequency; LF: low frequency. Source data are provided as a Source Data file.

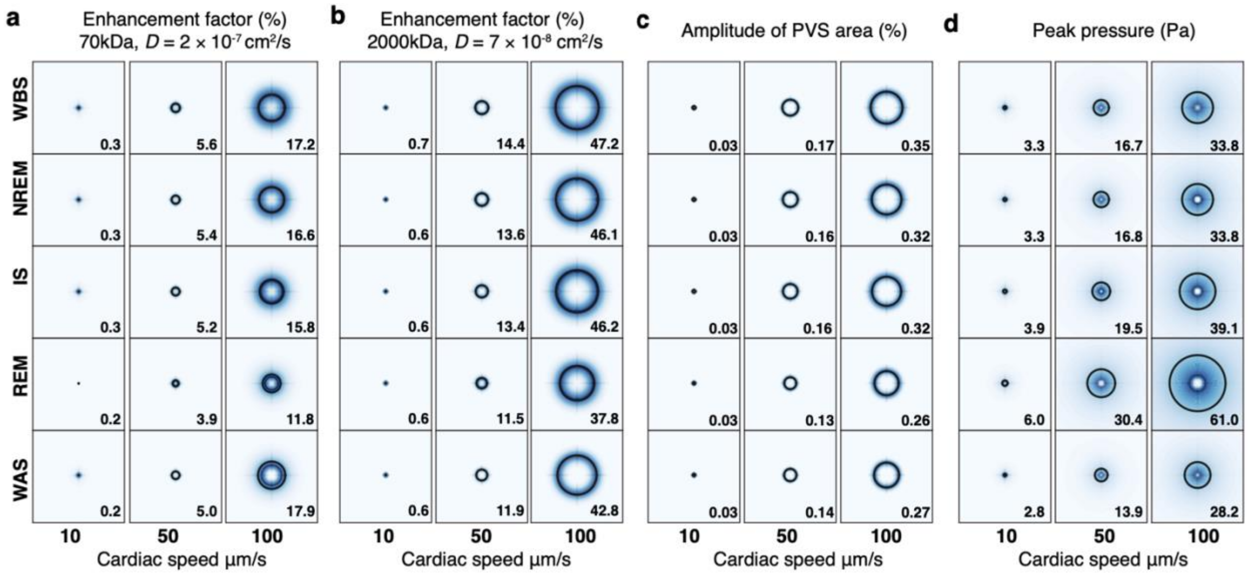

Supplementary Fig. 13: **Biomechanical modeling of CSF flow and solute transport in penetrating arteriole PVS generated by cardiac pulsations with different peak CSF velocities. a,** Enhancement factor for 70 kDa solutes, **b,** enhancement factor for 2000 kDa solutes, **c,** amplitude of PVS area, and **d,** peak pressure generated by cardiac pulsations with peak CSF velocity of 10, 50 or 100 µm/s across states of a sleep cycle as predicted by biomechanical modeling. The blue color surface represents the distribution obtained from all observations, the black line is the median of the observations. WBS: wake before sleep; NREM: non-rapid eye movement sleep; IS: intermediate state sleep; REM: rapid eye movement sleep; WAS: wake after sleep; PVS: perivascular space. Source data are provided as a Source Data file.

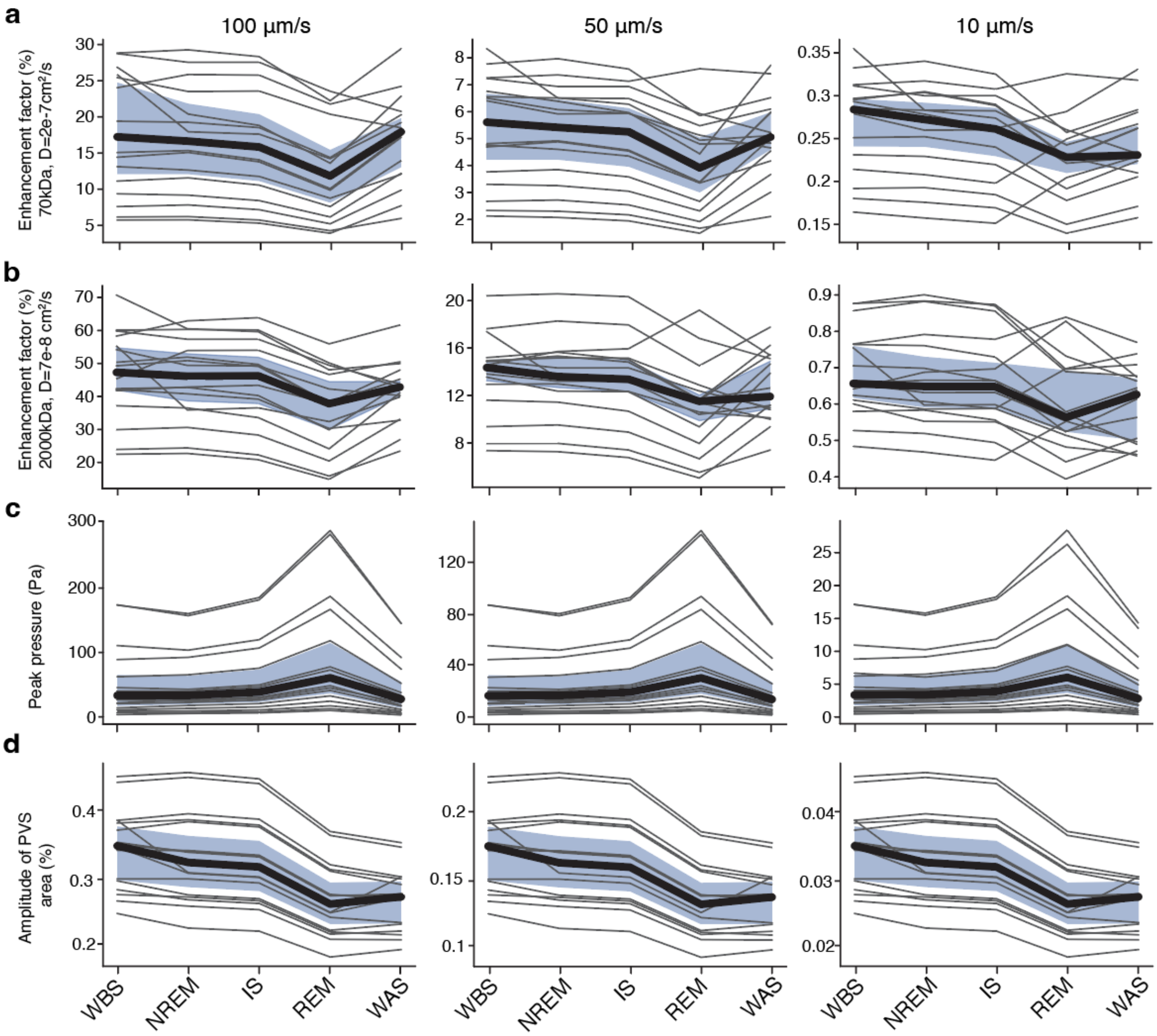

Supplementary Fig. 14: Biomechanical modeling of CSF flow and solute transport in penetrating arteriole PVS generated by cardiac pulsations with different peak CSF velocities. a, Enhancement factor for 70 kDa solutes, **b,** enhancement factor for 2000 kDa solutes, **c,** peak pressure**,** and **d,** amplitude of PVS area, generated by cardiac pulsations with peak CSF velocity of 100, 50 or 10 μm/s across the states of a sleep cycle as predicted by biomechanical modeling. Gray lines represent observations from individual penetrating arterioles while bolded black lines with the blue shading are median values with 10th and 90th percentiles, n = 16 vessels, 4 mice. WBS: wake before sleep; NREM: non-rapid eye movement sleep; IS: intermediate state sleep; REM: rapid eye movement sleep; WAS: wake after sleep; PVS: perivascular space. Source data are provided as a Source Data file.

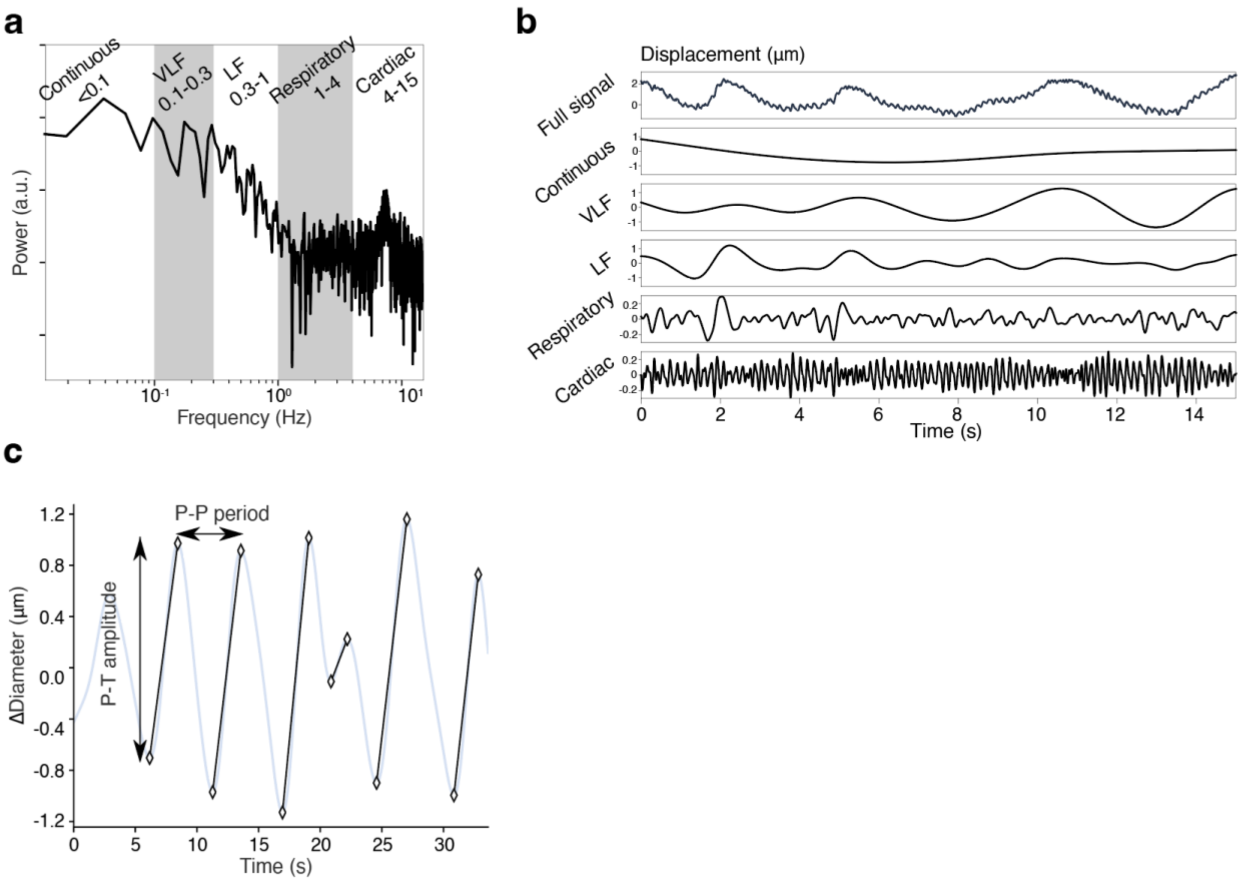

Supplementary Fig. 15: a, Power spectrum of vessel diameter oscillations and different frequency bands used for analysis. **b,** Signal decomposition into continuous, VLF, LF, respiratory and cardiac frequency bands. **c,** Amplitude of oscillations in different frequency bands was calculated as peak-to-trough (P-T), while period as peak-to-peak (P-P). VLF: very low frequency; LF: low frequency.

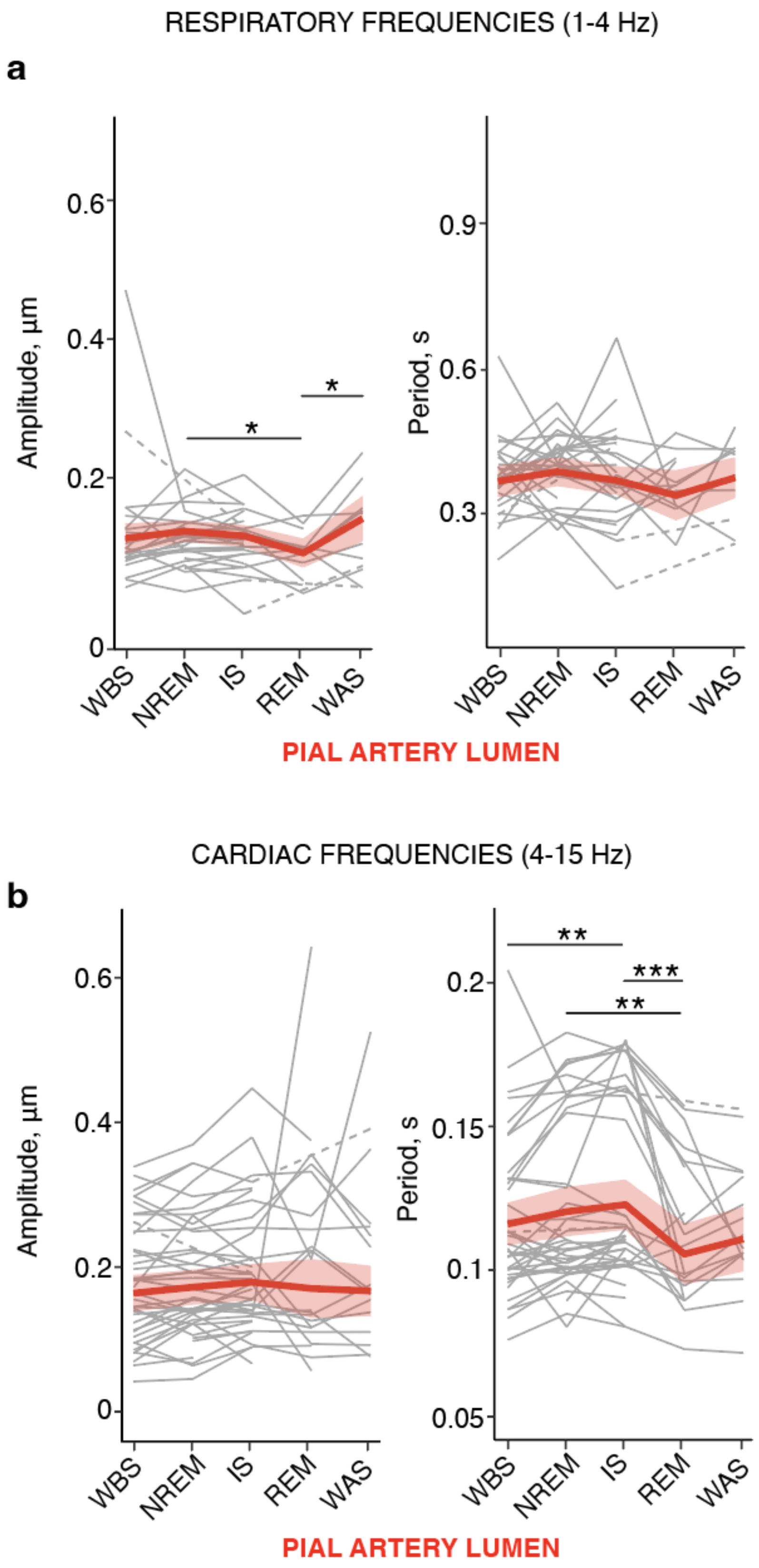
RESPIRATORY FREQUENCIES (1-4 Hz)
a
Amplitude, µm
0.6
0.4
0.2
0
*
*
Period, s
0.9
0.6
0.3
WBS
NREM
IS
REM
WAS
PIAL ARTERY LUMEN
CARDIAC FREQUENCIES (4-15 Hz)
b
Amplitude, µm
0.6
0.4
0.2
0
Period, s
0.2
0.15
0.1
0.05
**
***
**
WBS
NREM
IS
REM
WAS
PIAL ARTERY LUMEN

Supplementary Fig. 16: Amplitude and period of **a,** respiratory (1-4Hz) and **b,** cardiac (4-15Hz) frequencies of pial artery lumen throughout a sleep cycle. Gray lines represent the individual pial arteries, dashed lines indicate that a particular artery has no observations in a certain state, bold lines and shaded area are the estimates and 95% CI from linear mixed effects models (two-sided), n = 343 episodes, 30 pial arteries, 5 mice for **a**, n = 487 episodes, 43 pial arteries, 4 mice for **b**. $^*P < 0.05$, $^{**}P < 0.01$, $^{***}P < 0.001$, Tukey adjustment for multiple comparisons. WBS: wake before sleep; NREM: non-rapid eye movement sleep; IS: intermediate state sleep; REM: rapid eye movement sleep; WAS: wake after sleep. See end of supplementary information for exact p-values, and degrees of freedom for the statistical tests. Source data are provided as a Source Data file.

RESPIRATORY FREQUENCIES (1-4 Hz)

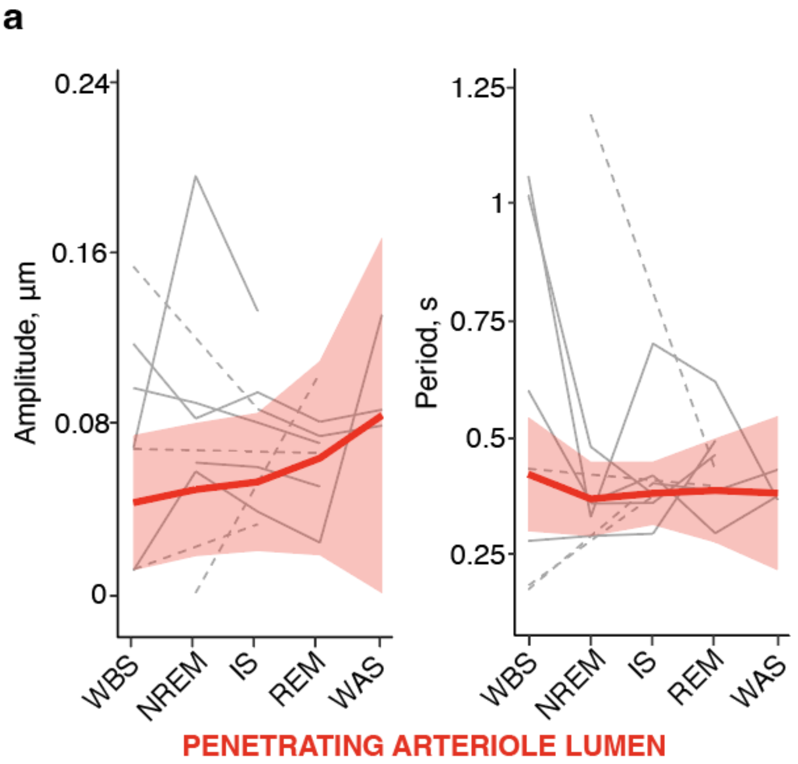

CARDIAC FREQUENCIES (4-15 Hz)

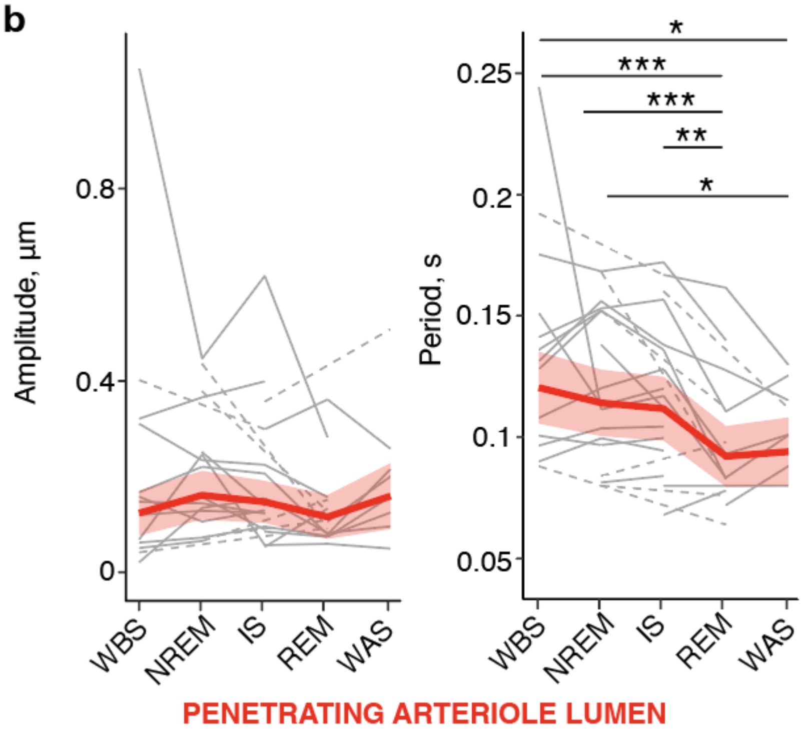

Supplementary Fig. 17: Amplitude and period of **a,** respiratory (1-4Hz) and **b,** cardiac (4-15Hz) frequencies of penetrating arteriole lumen throughout a sleep cycle. Gray lines represent the individual penetrating arterioles, dashed lines indicate that a particular penetrating arteriole has no observations in a certain state, bold lines and shaded area are the estimates and 95% CI from linear mixed effects models (two-sided), n=135 episodes, 22 penetrating arterioles, 4 mice for **a**, n=57 episodes, 9 penetrating arterioles, 3 mice for **b**. $*P < 0.05$, $**P < 0.01$, $***P < 0.001$, Tukey adjustment for multiple comparisons. WBS: wake before sleep; NREM: non-rapid eye movement sleep; IS: intermediate state sleep; REM: rapid eye movement sleep; WAS: wake after sleep. See end of supplementary information for exact p-values, and degrees of freedom for the statistical tests. Source data are provided as a Source Data file.

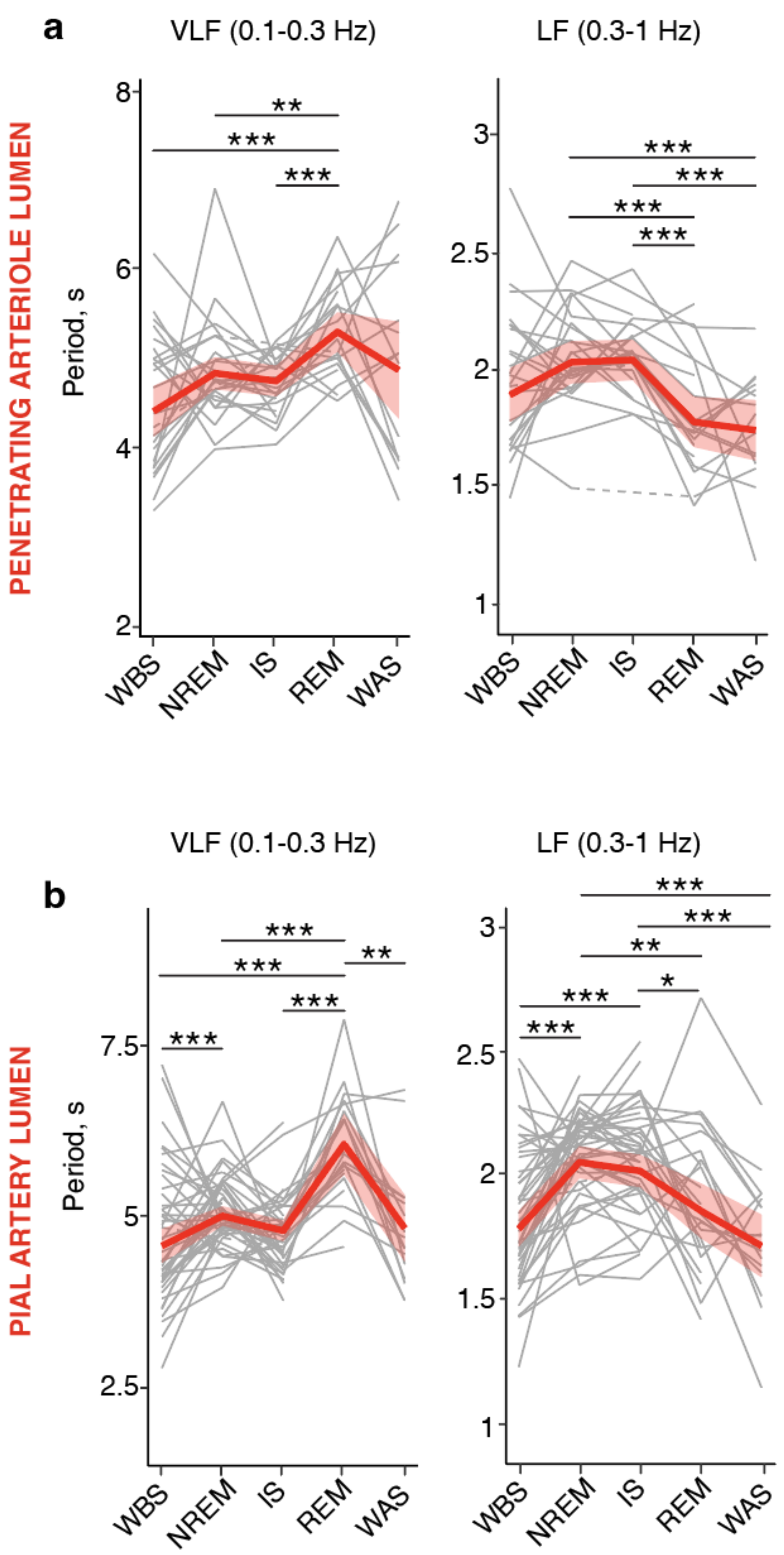
a
VLF (0.1-0.3 Hz)
LF (0.3-1 Hz)
PENETRATING ARTERIOLE LUMEN
Period, s
WBS
NREM
IS
REM
WAS
b
PIAL ARTERY LUMEN

Supplementary Fig. 18: Period of VLF and LF oscillations in lumen of **a,** pial arteries and **b,** penetrating arterioles throughout a sleep cycle. Gray lines represent the individual vessel, dashed lines indicate that a particular vessel has no observations in a certain state, bold lines and shaded area are the estimates and 95% CI from linear mixed effects models (two-sided), n=310 episodes, 25 penetrating arterioles, 4 mice for **a**, n=570 episodes, 44 penetrating arterioles, 5 mice for **b**. $*P < 0.05$, $**P < 0.01$, $***P < 0.001$, Tukey adjustment for multiple comparisons. WBS: wake before sleep; NREM: non-rapid eye movement sleep; IS: intermediate state sleep; REM: rapid eye movement sleep; WAS: wake after sleep; VLF: very low frequency; LF: low frequency. See end of supplementary information for exact p-values, and degrees of freedom for the statistical tests. Source data are provided as a Source Data file.

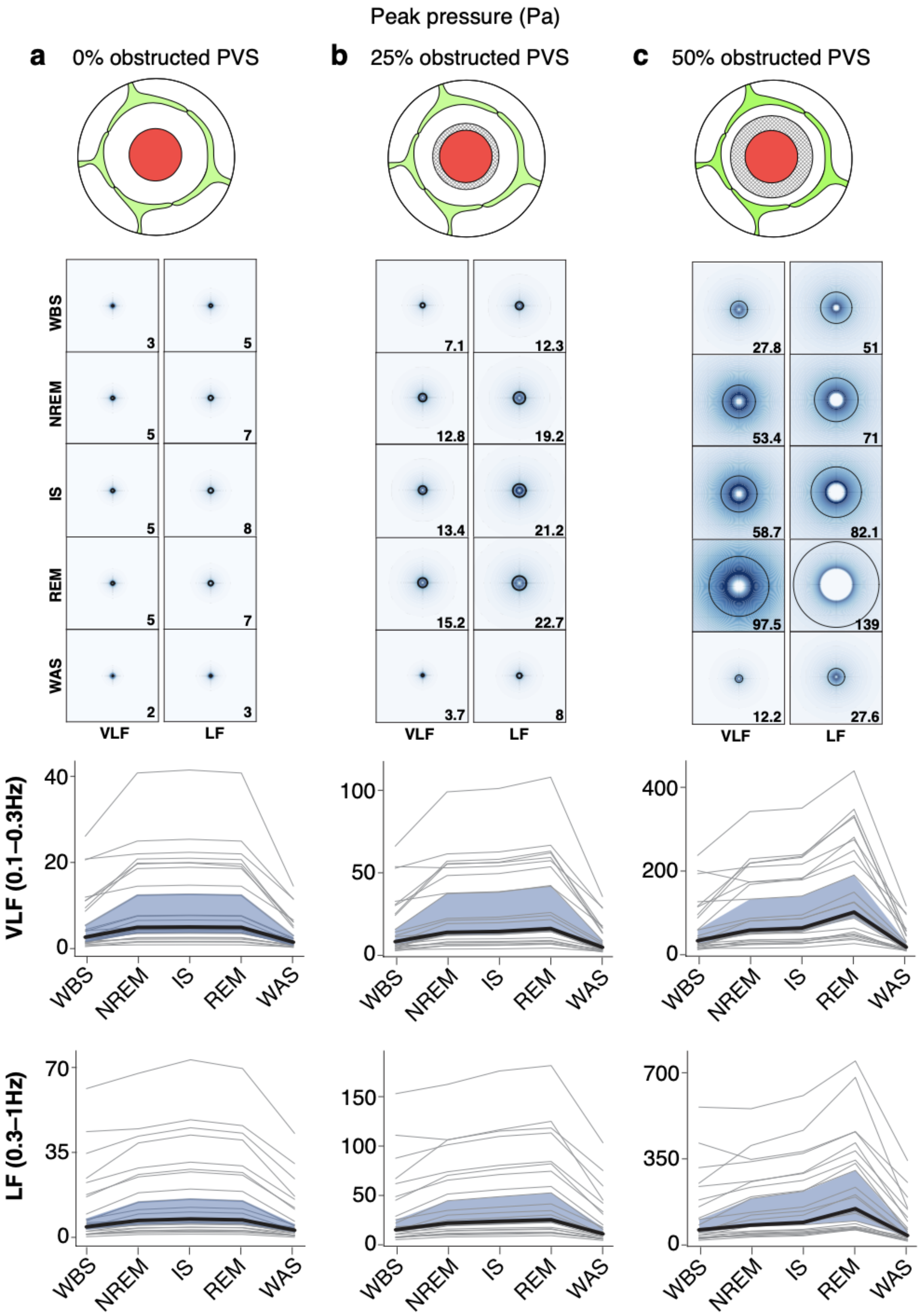

Peak pressure (Pa)
a
0% obstructed PVS
b
25% obstructed PVS
c
50% obstructed PVS
WBS
NREM
IS
REM
WAS
VLF
LF
3
5
5
7
5
8
5
7
2
3
7.1
12.3
12.8
19.2
13.4
21.2
15.2
22.7
3.7
8
27.8
51
53.4
71
58.7
82.1
97.5
139
12.2
27.6
VLF (0.1–0.3Hz)
LF (0.3–1Hz)

Supplementary Fig. 19. Predicted peak pressure generated by VLF and LF oscillations across sleep-wake cycle in penetrating arteriole PVS. a-c, Peak pressure in penetrating arteriole PVS generated by VLF and LF oscillations during a sleep cycle as predicted by biomechanical modeling using three scenarios where we added a fixed volume to the PVS obstructing flow corresponding to 0%, 25% and 50% of our measured PVS in quiet wakefulness. The black circle represents the median, whereas the shading represents the distribution of all modeled vessels. Gray lines represent observations from individual penetrating arterioles while bolded black lines with the blue shading are median values with 10th and 90th percentiles, n = 16 vessels, 4 mice. WBS: wake before sleep; NREM: non-rapid eye movement sleep; IS: intermediate state sleep; REM: rapid eye movement sleep; WAS: wake after sleep; PVS: perivascular space; VLF: very low frequency; LF: low frequency. See end of supplementary information for exact p-values, and degrees of freedom for the statistical tests. Source data are provided as a Source Data file.

Supplementary tables

**Lumen diameter**	Wake before sleep	NREM	IS	REM	Wake after sleep
VLF amplitude, µm (%)	0.4 (1.8)	1.4 (5.9)	1.2 (4.9)	0.9 (3.1)	0.7 (3.2)
LF amplitude, µm (%)	0.3 (1.3)	0.6 (2.5)	0.6 (2.5)	0.3 (1.0)	0.3 (1.4)
Median diameter, µm (%)	22.7	23.6 (4.0)	24.4 (7.5)	29.2 (28.6)	21.6(-4.8)

Supplementary Table 1. Estimated values of VLF amplitude, LF amplitude and diameter of pial artery lumen during sleep states obtained from linear mixed effects models. For VLF amplitude and LF amplitude, numbers in brackets show the percentage wise change in diameter from median diameter in each sleep cycle state. For median diameter, numbers in brackets show percentage wise change in diameter from wake-before-sleep state.

**Lumen diameter**	Quiet wakefulness	Whisking	Locomotion
VLF amplitude, µm (%)	0.6 (3.3)	0.4 (2.2)	1.4 (7.4)
LF amplitude, µm (%)	0.3 (1.7)	0.3 (1.7)	0.7 (3.7)
Median diameter, µm (%)	18	18.1 (0.6)	19.0 (5.6)

Supplementary Table 2. Estimated values of VLF amplitude, LF amplitude and diameter of pial artery lumen during wake states obtained from linear mixed effects models. For VLF amplitude and LF amplitude, numbers in brackets show the percentage wise change in diameter from median diameter in each wakefulness state. For median diameter, numbers in brackets show percentage wise change in diameter from quiet wakefulness state.

**Lumen diameter**	Wake before sleep	NREM	IS	REM	Wake after sleep
VLF amplitude, µm	0.4 (3.4)	1 (8.2)	0.8 (6.1)	0.4 (2.6)	0.3 (2.7)
LF amplitude, µm	0.3 (2.5)	0.5 (4.1)	0.45 (3.4)	0.2 (1.3)	0.2 (1.8)
Median diameter, µm	11.9	12.2 (2.5)	13.2 (10.9)	15.5 (30.3)	11.3 (-5.0)

Supplementary Table 3. Estimated values of VLF amplitude, LF amplitude and diameter of penetrating arteriole lumen during sleep states obtained from linear mixed effects models. For VLF amplitude and LF amplitude, numbers in brackets show the percentage wise change in diameter from median diameter in each sleep cycle state. For median diameter, numbers in brackets show percentage wise change in diameter from wake-before-sleep state.

**PVS total width**	Wake before sleep	NREM	IS	REM	Wake after sleep
VLF amplitude, µm (%)	0.29 (4.7)	0.44 (7.2)	0.4 (7.1)	0.21 (4.7)	0.16 (2.4)
LF amplitude, µm (%)	0.16 (2.6)	0.27 (4.4)	0.25 (4.5)	0.11 (2.4)	0.14 (2.1)
Median total width, µm (%)	6.2	6.1 (-1.6)	5.6 (-9.7)	4.5 (-27.4)	6.8 (9.7)

Supplementary Table 4. Estimated values of VLF amplitude, LF amplitude and total width of penetrating arteriole PVS during sleep states obtained from linear mixed effects models. For VLF amplitude and LF amplitude, numbers in brackets show the percentage wise change in diameter from median diameter in each sleep cycle state. For median total width, numbers in brackets show percentage wise change in total width from wake-before-sleep state.

**PVS area**	Wake before sleep	NREM	IS	REM	Wake after sleep
VLF amplitude, μm^2 (%)	5 (3.4)	8.7 (5.9)	7.5 (5.2)	4.8 (3.8)	3.7 (2.3)
LF amplitude, μm^2 (%)	3.2 (2.2)	5.4 (3.6)	4.8 (3.4)	2.2 (1.7)	2.8 (1.7)
Median area, μm^2 (%)	148	148 (0)	143 (-3.4)	126 (-14.9)	161 (8.8)

Supplementary Table 5. Estimated values of VLF amplitude, LF amplitude and median area of penetrating arteriole PVS during sleep states obtained from linear mixed effects models. For VLF amplitude and LF amplitude, numbers in brackets show the percentage wise change in area from median area in each sleep cycle state. For the median area, numbers in brackets show percentage wise change in area from wake-before-sleep state.

**Endfoot tube diameter**	Wake before sleep	NREM	IS	REM	Wake after sleep
VLF amplitude, µm (%)	0.19 (1.0)	0.37 (1.9)	0.33 (1.7)	0.21 (1.0)	0.15 (0.7)
LF amplitude, µm (%)	0.13 (0.7)	0.24 (1.3)	0.22 (1.1)	0.1 (0.5)	0.13 (0.6)
Median diameter, µm (%)	19	19.2 (1.1)	19.4 (2.1)	20.5 (7.9)	19.2 (1.1)

Supplementary Table 6. Estimated values of VLF amplitude, LF amplitude and diameter of penetrating arteriole endfoot tube during sleep states obtained from linear mixed effects models. For VLF amplitude and LF amplitude, numbers in brackets show the percentage wise change in diameter from median diameter in each sleep cycle state. For median diameter, numbers in brackets show percentage wise change in diameter from wake-before-sleep state.

**Lumen diameter**	Quiet wakefulness	Whisking	Locomotion
VLF amplitude, µm (%)	0.5 (4.4)	0.4 (3.3)	0.63 (4.7)
LF amplitude, µm (%)	0.37 (3.2)	0.31 (2.5)	0.46 (3.5)
Median diameter, µm (%)	11.4	12.3 (7.9)	13.3 (16.7)

Supplementary Table 7. Estimated values of VLF amplitude, LF amplitude and diameter of penetrating arteriole lumen during wake states obtained from linear mixed effects models. For VLF amplitude and LF amplitude, numbers in brackets show the percentage wise change in diameter from median diameter in each wakefulness state. For median diameter, numbers in brackets show percentage wise change in diameter from quiet wakefulness state.

**PVS total width**	Quiet wakefulness	Whisking	Locomotion
VLF amplitude, µm (%)	0.31 (4.8)	0.26 (4.3)	0.57 (10.4)
LF amplitude, µm (%)	0.23 (3.5)	0.24 (4.0)	0.44 (8.0)
Median total width, µm (%)	6.5	6 (-7.7)	5.5 (-15.4)

Supplementary Table 8. Estimated values of VLF amplitude, LF amplitude and total width of penetrating arteriole PVS during wake states obtained from linear mixed effects models. For VLF amplitude and LF amplitude, numbers in brackets show the percentage wise change in total width from median total width in each wakefulness state. For total width, numbers in brackets show percentage wise change in total width from quiet wakefulness state.

**PVS area**	Quiet wakefulness	Whisking	Locomotion
VLF amplitude, µm^2 (%)	5.7 (3.9)	5.2 (3.7)	10 (7.4)
LF amplitude, µm^2 (%)	3.9 (2.6)	4.9 (3.5)	10 (7.4)
Median area, µm^2 (%)	148	142 (-4.1)	136 (-8.1)

Supplementary Table 9. Estimated values of VLF amplitude, LF amplitude and median area of penetrating arteriole PVS during wake states obtained from linear mixed effects models. For VLF amplitude and LF amplitude, numbers in brackets show the percentage wise change in area from

median area in each wakefulness state. For the median area, numbers in brackets show percentage wise change in area from quiet wakefulness state.

**Endfoot tube diameter**	Quiet wakefulness	Whisking	Locomotion
VLF amplitude, µm (%)	0.13 (0.7)	0.18 (1.0)	0.3 (1.6)
LF amplitude, µm (%)	0.13 (0.7)	0.14 (0.8)	0.32 (1.7)
Median diameter, µm (%)	18.3	18.6 (1.6)	19 (3.8)

Supplementary Table 10. Estimated values of VLF amplitude, LF amplitude and diameter of penetrating arteriole endfoot tube during wake states obtained from linear mixed effects models. For VLF amplitude and LF amplitude, numbers in brackets show the percentage wise change in diameter from median diameter in each wakefulness state. For median diameter, numbers in brackets show percentage wise change in diameter from quiet wakefulness state.

STATISTICAL TEST DETAILS
Effect sizes, degrees of freedom and p-values

Figure 1

1 d) Pial artery, VLF, amplitude

	contrast	ratio	SE	df	t.ratio	p.value
1	NREM / Quiet	3.192	0.351	551	10.549	< 0.0001
2	IS / Quiet	2.862	0.322	551	9.356	< 0.0001
3	IS / NREM	0.896	0.039	551	-2.487	0.095
4	REM / Quiet	2.012	0.365	551	3.857	0.001
5	REM / NREM	0.630	0.092	551	-3.152	0.015
6	REM / IS	0.703	0.101	551	-2.464	0.101
7	Awakening / Quiet	1.547	0.404	551	1.669	0.454
8	Awakening / NREM	0.485	0.130	551	-2.692	0.056
9	Awakening / IS	0.541	0.146	551	-2.278	0.154
10	Awakening / REM	0.769	0.235	551	-0.862	0.911

1 e) Pial artery, LF, amplitude

	contrast	ratio	SE	df	t.ratio	p.value
1	NREM / Quiet	2.141	0.134	560	12.157	< 0.0001
2	IS / Quiet	2.103	0.136	560	11.517	< 0.0001
3	IS / NREM	0.982	0.030	560	-0.594	0.976
4	REM / Quiet	0.960	0.124	560	-0.318	0.998
5	REM / NREM	0.448	0.051	560	-6.999	< 0.0001
6	REM / IS	0.456	0.051	560	-6.958	< 0.0001
7	Awakening / Quiet	0.995	0.186	560	-0.028	1.000
8	Awakening / NREM	0.465	0.088	560	-4.062	0.001
9	Awakening / IS	0.473	0.090	560	-3.950	0.001
10	Awakening / REM	1.037	0.227	560	0.164	1.000

Tables in Latex format generated by `Hlavac, Marek (2022). stargazer: Well-Formatted Regression and Summary Statistics Tables. R package version 5.2.3. https://CRAN.R-project.org/package=stargazer`

1 f) Pial artery, median diameter

	contrast	ratio	SE	df	t.ratio	p.value
1	NREM / Quiet	1.041	0.011	562	3.912	0.001
2	IS / Quiet	1.069	0.011	562	6.370	< 0.0001
3	IS / NREM	1.026	0.004	562	6.286	< 0.0001
4	REM / Quiet	1.283	0.031	562	10.243	< 0.0001
5	REM / NREM	1.233	0.028	562	9.316	< 0.0001
6	REM / IS	1.201	0.027	562	8.216	< 0.0001
7	Awakening / Quiet	0.961	0.020	562	-1.879	0.330
8	Awakening / NREM	0.923	0.021	562	-3.499	0.005
9	Awakening / IS	0.900	0.021	562	-4.623	0.00005
10	Awakening / REM	0.749	0.024	562	-9.076	< 0.0001

Figure 2

2 d) Penetrating arteriole, VLF, amplitude, Lumen

	contrast	ratio	SE	df	t.ratio	p.value
1	NREM / Quiet	2.214	0.260	290	6.757	< 0.0001
2	IS / Quiet	1.834	0.219	290	5.092	< 0.0001
3	IS / NREM	0.828	0.044	290	-3.544	0.004
4	REM / Quiet	0.807	0.142	290	-1.216	0.742
5	REM / NREM	0.365	0.050	290	-7.385	< 0.0001
6	REM / IS	0.440	0.057	290	-6.354	< 0.0001
7	Awakening / Quiet	0.618	0.104	290	-2.857	0.037
8	Awakening / NREM	0.279	0.045	290	-7.863	< 0.0001
9	Awakening / IS	0.337	0.055	290	-6.641	< 0.0001
10	Awakening / REM	0.765	0.159	290	-1.293	0.696

2 d) Penetrating arteriole, VLF, amplitude, PVS

	contrast	ratio	SE	df	t.ratio	p.value
1	NREM / Quiet	1.526	0.197	285	3.277	0.010
2	IS / Quiet	1.385	0.179	285	2.528	0.087
3	IS / NREM	0.907	0.055	285	-1.605	0.495
4	REM / Quiet	0.745	0.114	285	-1.916	0.311
5	REM / NREM	0.488	0.050	285	-6.939	< 0.0001
6	REM / IS	0.538	0.048	285	-6.951	< 0.0001
7	Awakening / Quiet	0.559	0.093	285	-3.487	0.005
8	Awakening / NREM	0.366	0.059	285	-6.286	< 0.0001
9	Awakening / IS	0.404	0.066	285	-5.571	< 0.0001
10	Awakening / REM	0.750	0.139	285	-1.556	0.527

2 e) Penetrating arteriole, median diameter, Lumen

	contrast	ratio	SE	df	t.ratio	p.value
1	NREM / Quiet	1.035	0.023	294	1.554	0.528
2	IS / Quiet	1.116	0.023	294	5.290	< 0.0001
3	IS / NREM	1.079	0.012	294	6.991	< 0.0001
4	REM / Quiet	1.310	0.045	294	7.896	< 0.0001
5	REM / NREM	1.266	0.037	294	8.021	< 0.0001
6	REM / IS	1.173	0.033	294	5.628	< 0.0001
7	Awakening / Quiet	0.952	0.024	294	-1.929	0.304
8	Awakening / NREM	0.920	0.030	294	-2.598	0.073
9	Awakening / IS	0.853	0.027	294	-5.090	< 0.0001
10	Awakening / REM	0.727	0.030	294	-7.755	< 0.0001

2 e) Penetrating arteriole, median diameter, PVS

Table 1:

	contrast	ratio	SE	df	t.ratio	p.value
1	NREM / Quiet	0.979	0.024	294	-0.853	0.914
2	IS / Quiet	0.911	0.022	294	-3.849	0.001
3	IS / NREM	0.930	0.012	294	-5.474	< 0.0001
4	REM / Quiet	0.730	0.034	294	-6.858	< 0.0001
5	REM / NREM	0.745	0.031	294	-7.123	< 0.0001
6	REM / IS	0.801	0.032	294	-5.485	< 0.0001
7	Awakening / Quiet	1.093	0.032	294	3.001	0.024
8	Awakening / NREM	1.116	0.036	294	3.407	0.007
9	Awakening / IS	1.200	0.038	294	5.729	< 0.0001
10	Awakening / REM	1.498	0.074	294	8.138	< 0.0001

Supplementary Figure 2

a) Pial arteries, wakefullness, VLF, amplitude, Lumen

	contrast	ratio	SE	df	t.ratio	p.value
1	Whisking / Quiet	0.768	0.340	483	-0.596	0.822
2	Locomotion / Quiet	2.520	0.360	483	6.468	< 0.0001
3	Locomotion / Whisking	3.280	1.445	483	2.696	0.020

b) Pial arteries, wakefullness, LF, amplitude, Lumen

	contrast	ratio	SE	df	t.ratio	p.value
1	Whisking / Quiet	0.997	0.190	782	-0.018	1.000
2	Locomotion / Quiet	3.512	0.553	782	7.983	< 0.0001
3	Locomotion / Whisking	3.524	0.656	782	6.765	< 0.0001

c) Pial arteries, wakefullness, median diameter, Lumen

	contrast	ratio	SE	df	t.ratio	p.value
1	Whisking / Quiet	1.003	0.018	875	0.184	0.981
2	Locomotion / Quiet	1.054	0.018	875	3.099	0.006
3	Locomotion / Whisking	1.051	0.016	875	3.313	0.003

Supplementary Figure 3

a) Penetrating arteriole, VLF, amplitude, Endfeet

	contrast	ratio	SE	df	t.ratio	p.value
1	NREM / Quiet	1.920	0.175	292	7.155	< 0.0001
2	IS / Quiet	1.732	0.163	292	5.827	< 0.0001
3	IS / NREM	0.902	0.060	292	-1.546	0.533
4	REM / Quiet	1.089	0.162	292	0.570	0.979
5	REM / NREM	0.567	0.074	292	-4.320	0.0002
6	REM / IS	0.629	0.078	292	-3.743	0.002
7	Awakening / Quiet	0.775	0.111	292	-1.775	0.390
8	Awakening / NREM	0.403	0.053	292	-6.905	< 0.0001
9	Awakening / IS	0.447	0.060	292	-5.966	< 0.0001
10	Awakening / REM	0.711	0.126	292	-1.929	0.304

b) Penetrating arteriole, median diameter, Endfeet

	contrast	ratio	SE	df	t.ratio	p.value
1	NREM / Quiet	1.010	0.006	294	1.778	0.388
2	IS / Quiet	1.025	0.006	294	4.301	0.0002
3	IS / NREM	1.014	0.003	294	4.902	< 0.0001
4	REM / Quiet	1.081	0.017	294	5.112	< 0.0001
5	REM / NREM	1.070	0.016	294	4.654	< 0.0001
6	REM / IS	1.055	0.015	294	3.730	0.002
7	Awakening / Quiet	1.013	0.011	294	1.106	0.803
8	Awakening / NREM	1.002	0.012	294	0.176	1.000
9	Awakening / IS	0.988	0.012	294	-0.968	0.869
10	Awakening / REM	0.936	0.017	294	-3.617	0.003

Supplementary Figure 4

a) Penetrating arteriole, LF, amplitude, Endfeet

	contrast	ratio	SE	df	t.ratio	p.value
1	NREM / Quiet	1.823	0.202	295	5.409	< 0.0001
2	IS / Quiet	1.682	0.187	295	4.667	< 0.0001
3	IS / NREM	0.923	0.036	295	-2.081	0.231
4	REM / Quiet	0.785	0.135	295	-1.409	0.622
5	REM / NREM	0.431	0.058	295	-6.207	< 0.0001
6	REM / IS	0.467	0.061	295	-5.803	< 0.0001
7	Awakening / Quiet	0.984	0.138	295	-0.118	1.000
8	Awakening / NREM	0.540	0.064	295	-5.189	< 0.0001
9	Awakening / IS	0.585	0.071	295	-4.439	< 0.0001
10	Awakening / REM	1.253	0.225	295	1.251	0.721

b) Penetrating arteriole, LF, amplitude, Lumen

	contrast	ratio	SE	df	t.ratio	p.value
1	NREM / Quiet	1.853	0.177	292	6.444	< 0.0001
2	IS / Quiet	1.669	0.161	292	5.297	< 0.0001
3	IS / NREM	0.901	0.038	292	-2.479	0.098
4	REM / Quiet	0.611	0.089	292	-3.362	0.008
5	REM / NREM	0.330	0.038	292	-9.585	< 0.0001
6	REM / IS	0.366	0.039	292	-9.424	< 0.0001
7	Awakening / Quiet	0.811	0.121	292	-1.400	0.628
8	Awakening / NREM	0.438	0.059	292	-6.128	< 0.0001
9	Awakening / IS	0.486	0.065	292	-5.362	< 0.0001
10	Awakening / REM	1.327	0.224	292	1.679	0.449

c) Penetrating arteriole, LF, amplitude, PVS

	contrast	ratio	SE	df	t.ratio	p.value
1	NREM / Quiet	1.681	0.172	292	5.058	< 0.0001
2	IS / Quiet	1.535	0.162	292	4.052	0.001
3	IS / NREM	0.914	0.039	292	-2.144	0.205
4	REM / Quiet	0.677	0.095	292	-2.778	0.046
5	REM / NREM	0.403	0.041	292	-8.983	< 0.0001
6	REM / IS	0.441	0.041	292	-8.739	< 0.0001
7	Awakening / Quiet	0.849	0.131	292	-1.065	0.824
8	Awakening / NREM	0.505	0.074	292	-4.662	< 0.0001
9	Awakening / IS	0.553	0.082	292	-3.989	0.001
10	Awakening / REM	1.255	0.220	292	1.293	0.696

Supplementary Figure 5

a) Penetrating arteriole, median area

	contrast	ratio	SE	df	t.ratio	p.value
1	NREM / Quiet	1.003	0.018	294	0.145	1.000
2	IS / Quiet	0.965	0.017	294	-2.012	0.263
3	IS / NREM	0.962	0.011	294	-3.417	0.006
4	REM / Quiet	0.851	0.037	294	-3.737	0.002
5	REM / NREM	0.848	0.035	294	-4.024	0.001
6	REM / IS	0.882	0.036	294	-3.116	0.017
7	Awakening / Quiet	1.089	0.033	294	2.849	0.038
8	Awakening / NREM	1.087	0.032	294	2.854	0.037
9	Awakening / IS	1.129	0.033	294	4.178	0.0004
10	Awakening / REM	1.281	0.061	294	5.203	< 0.0001

b) Penetrating arteriole, LF, amplitude area

	contrast	ratio	SE	df	t.ratio	p.value
1	NREM / Quiet	1.666	0.172	292	4.952	< 0.0001
2	IS / Quiet	1.471	0.152	292	3.724	0.002
3	IS / NREM	0.883	0.038	292	-2.879	0.034
4	REM / Quiet	0.688	0.090	292	-2.857	0.037
5	REM / NREM	0.413	0.038	292	-9.642	< 0.0001
6	REM / IS	0.467	0.040	292	-8.975	< 0.0001
7	Awakening / Quiet	0.889	0.138	292	-0.758	0.942
8	Awakening / NREM	0.534	0.073	292	-4.601	0.0001
9	Awakening / IS	0.604	0.084	292	-3.616	0.003
10	Awakening / REM	1.293	0.210	292	1.579	0.512

c) Penetrating arteriole, VLF, amplitude area

	contrast	ratio	SE	df	t.ratio	p.value
1	NREM / Quiet	1.720	0.234	289	3.981	0.001
2	IS / Quiet	1.480	0.205	289	2.833	0.039
3	IS / NREM	0.860	0.051	289	-2.526	0.088
4	REM / Quiet	0.950	0.169	289	-0.291	0.998
5	REM / NREM	0.552	0.070	289	-4.657	< 0.0001
6	REM / IS	0.642	0.076	289	-3.730	0.002
7	Awakening / Quiet	0.740	0.157	289	-1.418	0.617
8	Awakening / NREM	0.430	0.084	289	-4.297	0.0002
9	Awakening / IS	0.500	0.100	289	-3.470	0.005
10	Awakening / REM	0.779	0.180	289	-1.077	0.818

Supplementary Figure 6

a) Penetrating arteriole, wakefullness, VLF, amplitude

Endfeet

	contrast	ratio	SE	df	t.ratio	p.value
1	Whisking / Quiet	1.378	1.037	114	0.426	0.905
2	Locomotion / Quiet	2.274	0.347	114	5.382	< 0.0001
3	Locomotion / Whisking	1.650	1.236	114	0.669	0.782

Lumen

	contrast	ratio	SE	df	t.ratio	p.value
1	Whisking / Quiet	0.822	0.160	114	-1.009	0.573
2	Locomotion / Quiet	1.283	0.256	114	1.248	0.428
3	Locomotion / Whisking	1.561	0.346	114	2.011	0.114

PVS

	contrast	ratio	SE	df	t.ratio	p.value
1	Whisking / Quiet	0.822	0.095	123	-1.698	0.210
2	Locomotion / Quiet	1.820	0.209	123	5.210	< 0.0001
3	Locomotion / Whisking	2.215	0.286	123	6.160	< 0.0001

b) Penetrating arteriole, wakefullness, LF, amplitude

Endfeet

	contrast	ratio	SE	df	t.ratio	p.value
1	Whisking / Quiet	1.094	0.184	225	0.536	0.854
2	Locomotion / Quiet	2.467	0.217	225	10.283	< 0.0001
3	Locomotion / Whisking	2.254	0.385	225	4.760	< 0.0001

Lumen

	contrast	ratio	SE	df	t.ratio	p.value
1	Whisking / Quiet	0.844	0.162	215	-0.882	0.652
2	Locomotion / Quiet	1.241	0.200	215	1.340	0.375
3	Locomotion / Whisking	1.469	0.239	215	2.361	0.050

PVS

	contrast	ratio	SE	df	t.ratio	p.value
1	Whisking / Quiet	1.065	0.178	231	0.375	0.925
2	Locomotion / Quiet	1.942	0.241	231	5.343	< 0.0001
3	Locomotion / Whisking	1.824	0.272	231	4.024	0.0002

c) Penetrating arteriole, wakefullness, median diameter

Endfeet

	contrast	ratio	SE	df	t.ratio	p.value
1	Whisking / Quiet	1.014	0.010	251	1.387	0.349
2	Locomotion / Quiet	1.041	0.009	251	4.699	< 0.0001
3	Locomotion / Whisking	1.026	0.007	251	4.055	0.0002

Lumen

	contrast	ratio	SE	df	t.ratio	p.value
1	Whisking / Quiet	1.071	0.031	251	2.382	0.047
2	Locomotion / Quiet	1.167	0.027	251	6.673	< 0.0001
3	Locomotion / Whisking	1.090	0.022	251	4.334	< 0.0001

PVS

	contrast	ratio	SE	df	t.ratio	p.value
1	Whisking / Quiet	0.917	0.034	251	-2.334	0.053
2	Locomotion / Quiet	0.841	0.027	251	-5.453	< 0.0001
3	Locomotion / Whisking	0.917	0.023	251	-3.398	0.002

d) Penetrating arteriole, wakefullness, PVS area

VLF

	contrast	ratio	SE	df	t.ratio	p.value
1	Whisking / Quiet	0.907	0.187	123	-0.475	0.883
2	Locomotion / Quiet	1.829	0.240	123	4.611	< 0.0001
3	Locomotion / Whisking	2.017	0.439	123	3.225	0.005

LF

	contrast	ratio	SE	df	t.ratio	p.value
1	Whisking / Quiet	1.230	0.173	230	1.472	0.306
2	Locomotion / Quiet	2.563	0.289	230	8.344	< 0.0001
3	Locomotion / Whisking	2.084	0.281	230	5.440	< 0.0001

Median area

	contrast	ratio	SE	df	t.ratio	p.value
1	Whisking / Quiet	0.955	0.031	251	-1.431	0.327
2	Locomotion / Quiet	0.914	0.026	251	-3.178	0.005
3	Locomotion / Whisking	0.957	0.020	251	-2.146	0.083

Supplementary Figure 7

a) Veins, VLF, amplitude

Endfeet

	contrast	ratio	SE	df	t.ratio	p.value
1	NREM / Quiet	1.480	0.268	76	2.161	0.206
2	IS / Quiet	1.234	0.220	76	1.176	0.765
3	IS / NREM	0.834	0.100	76	-1.523	0.551
4	REM / Quiet	1.237	0.272	76	0.968	0.869
5	REM / NREM	0.836	0.155	76	-0.964	0.870
6	REM / IS	1.003	0.183	76	0.016	1
7	Awakening / Quiet	0.906	0.303	76	-0.294	0.998
8	Awakening / NREM	0.613	0.211	76	-1.421	0.616
9	Awakening / IS	0.735	0.250	76	-0.904	0.894
10	Awakening / REM	0.733	0.275	76	-0.830	0.921

Lumen

	contrast	ratio	SE	df	t.ratio	p.value
1	NREM / Quiet	1.465	0.281	75	1.989	0.281
2	IS / Quiet	1.020	0.184	75	0.112	1.000
3	IS / NREM	0.696	0.076	75	-3.305	0.012
4	REM / Quiet	0.980	0.225	75	-0.090	1.000
5	REM / NREM	0.669	0.161	75	-1.670	0.458
6	REM / IS	0.960	0.217	75	-0.181	1.000
7	Awakening / Quiet	0.756	0.244	75	-0.869	0.907
8	Awakening / NREM	0.516	0.153	75	-2.229	0.180
9	Awakening / IS	0.741	0.214	75	-1.037	0.837
10	Awakening / REM	0.772	0.264	75	-0.757	0.942

PVS

	contrast	ratio	SE	df	t.ratio	p.value
1	NREM / Quiet	1.395	0.244	73	1.903	0.325
2	IS / Quiet	1.243	0.217	73	1.248	0.724
3	IS / NREM	0.891	0.096	73	-1.068	0.822
4	REM / Quiet	1.151	0.291	73	0.555	0.981
5	REM / NREM	0.825	0.193	73	-0.822	0.923
6	REM / IS	0.925	0.204	73	-0.352	0.997
7	Awakening / Quiet	0.879	0.336	73	-0.337	0.997
8	Awakening / NREM	0.630	0.230	73	-1.268	0.711
9	Awakening / IS	0.707	0.255	73	-0.963	0.871
10	Awakening / REM	0.764	0.308	73	-0.667	0.963

b) Veins, LF, amplitude

Endfeet

	contrast	ratio	SE	df	t.ratio	p.value
1	NREM / Quiet	1.101	0.184	77	0.572	0.979
2	IS / Quiet	1.015	0.172	77	0.087	1.000
3	IS / NREM	0.922	0.076	77	-0.986	0.861
4	REM / Quiet	0.718	0.136	77	-1.750	0.410
5	REM / NREM	0.652	0.086	77	-3.235	0.015
6	REM / IS	0.707	0.097	77	-2.524	0.096
7	Awakening / Quiet	1.311	0.358	77	0.991	0.859
8	Awakening / NREM	1.191	0.347	77	0.599	0.975
9	Awakening / IS	1.292	0.374	77	0.883	0.902
10	Awakening / REM	1.826	0.566	77	1.944	0.303

Lumen

	contrast	ratio	SE	df	t.ratio	p.value
1	NREM / Quiet	1.167	0.112	77	1.608	0.497
2	IS / Quiet	1.084	0.104	77	0.841	0.917
3	IS / NREM	0.928	0.069	77	-1.008	0.851
4	REM / Quiet	0.832	0.106	77	-1.450	0.598
5	REM / NREM	0.712	0.088	77	-2.743	0.057
6	REM / IS	0.767	0.096	77	-2.108	0.227
7	Awakening / Quiet	0.619	0.128	77	-2.325	0.148
8	Awakening / NREM	0.531	0.108	77	-3.125	0.021
9	Awakening / IS	0.572	0.115	77	-2.789	0.051
10	Awakening / REM	0.745	0.164	77	-1.336	0.670

PVS

	contrast	ratio	SE	df	t.ratio	p.value
1	NREM / Quiet	0.946	0.168	73	-0.311	0.998
2	IS / Quiet	0.806	0.144	73	-1.205	0.748
3	IS / NREM	0.852	0.055	73	-2.483	0.106
4	REM / Quiet	0.687	0.130	73	-1.988	0.282
5	REM / NREM	0.726	0.067	73	-3.492	0.007
6	REM / IS	0.853	0.083	73	-1.644	0.475
7	Awakening / Quiet	1.019	0.138	73	0.141	1.000
8	Awakening / NREM	1.077	0.212	73	0.377	0.996
9	Awakening / IS	1.265	0.250	73	1.190	0.757
10	Awakening / REM	1.483	0.309	73	1.891	0.331

c) Veins, median diameter

Endfeet

	contrast	ratio	SE	df	t.ratio	p.value
1	NREM / Quiet	1.010	0.013	75	0.783	0.935
2	IS / Quiet	1.026	0.014	75	1.893	0.330
3	IS / NREM	1.016	0.006	75	2.521	0.097
4	REM / Quiet	1.003	0.020	75	0.172	1.000
5	REM / NREM	0.993	0.015	75	-0.449	0.991
6	REM / IS	0.978	0.015	75	-1.458	0.593
7	Awakening / Quiet	0.980	0.015	75	-1.343	0.665
8	Awakening / NREM	0.970	0.018	75	-1.610	0.496
9	Awakening / IS	0.955	0.019	75	-2.384	0.131
10	Awakening / REM	0.976	0.024	75	-0.981	0.863

Lumen

	contrast	ratio	SE	df	t.ratio	p.value
1	NREM / Quiet	1.030	0.020	75	1.529	0.547
2	IS / Quiet	1.054	0.019	75	2.939	0.034
3	IS / NREM	1.023	0.011	75	2.225	0.182
4	REM / Quiet	0.952	0.028	75	-1.652	0.470
5	REM / NREM	0.925	0.023	75	-3.188	0.017
6	REM / IS	0.904	0.021	75	-4.340	0.0004
7	Awakening / Quiet	0.998	0.031	75	-0.062	1.000
8	Awakening / NREM	0.969	0.032	75	-0.953	0.875
9	Awakening / IS	0.947	0.030	75	-1.694	0.444
10	Awakening / REM	1.048	0.041	75	1.205	0.748

PVS

	contrast	ratio	SE	df	t.ratio	p.value
1	NREM / Quiet	0.966	0.076	75	-0.446	0.992
2	IS / Quiet	0.948	0.074	75	-0.688	0.958
3	IS / NREM	0.982	0.021	75	-0.861	0.910
4	REM / Quiet	0.881	0.082	75	-1.374	0.646
5	REM / NREM	0.912	0.047	75	-1.771	0.398
6	REM / IS	0.929	0.048	75	-1.435	0.607
7	Awakening / Quiet	0.812	0.101	75	-1.671	0.458
8	Awakening / NREM	0.841	0.119	75	-1.220	0.740
9	Awakening / IS	0.857	0.121	75	-1.092	0.810
10	Awakening / REM	0.922	0.138	75	-0.539	0.983

Supplementary Figure 8

b) 0.1–0.3 Hz power, µm²

Endfoot

	contrast	p. value
1	NREM / IS	0.112
2	NREM / REM	0.016
3	NREM / quiet	0.017
4	IS / REM	0.058
5	IS / quiet	0.372
6	REM / quiet	0.338

Lumen

	contrast	p. value
1	NREM / IS	0.081
2	NREM / REM	0.072
3	NREM / quiet	0.02
4	IS / REM	0.175
5	IS / quiet	0.282
6	REM / quiet	0.422

PVS

	contrast	p. value
1	NREM / IS	0.34
2	NREM / REM	0.038
3	NREM / quiet	0.017
4	IS / REM	0.267
5	IS / quiet	0.453
6	REM / quiet	0.516

c) Change in diameter, µm

Endfoot

	contrast	p. value
1	NREM / IS	0.012
2	NREM / REM	<0.0001
3	NREM / quiet	0.035
4	IS / REM	<0.0001
5	IS / quiet	0.0006
6	REM / quiet	<0.0001

Lumen

	contrast	p. value
1	NREM / IS	0.029
2	NREM / REM	<0.0001
3	NREM / quiet	0.015
4	IS / REM	<0.0001
5	IS / quiet	0.0005
6	REM / quiet	<0.0001

PVS

	contrast	p. value
1	NREM / IS	0.066
2	NREM / REM	<0.0001
3	NREM / quiet	0.11
4	IS / REM	<0.0001
5	IS / quiet	0.0006
6	REM / quiet	<0.0001

Supplementary Figure 16

a) Pial artery, respiratory, Lumen

Amplitude

	contrast	ratio	SE	df	t.ratio	p.value
1	NREM / Quiet	1.091	0.085	324	1.118	0.797
2	IS / Quiet	1.031	0.084	324	0.372	0.996
3	IS / NREM	0.945	0.038	324	-1.400	0.628
4	REM / Quiet	0.812	0.100	324	-1.697	0.437
5	REM / NREM	0.744	0.074	324	-2.986	0.025
6	REM / IS	0.787	0.079	324	-2.385	0.122
7	Awakening / Quiet	1.251	0.150	324	1.861	0.341
8	Awakening / NREM	1.147	0.115	324	1.361	0.653
9	Awakening / IS	1.214	0.124	324	1.891	0.324
10	Awakening / REM	1.541	0.208	324	3.211	0.013

Period

	contrast	ratio	SE	df	t.ratio	p.value
1	NREM / Quiet	1.055	0.041	324	1.380	0.641
2	IS / Quiet	1.004	0.041	324	0.111	1.000
3	IS / NREM	0.953	0.025	324	-1.887	0.326
4	REM / Quiet	0.921	0.072	324	-1.057	0.828
5	REM / NREM	0.873	0.061	324	-1.952	0.292
6	REM / IS	0.917	0.064	324	-1.252	0.721
7	Awakening / Quiet	1.020	0.054	324	0.370	0.996
8	Awakening / NREM	0.967	0.047	324	-0.694	0.958
9	Awakening / IS	1.015	0.050	324	0.308	0.998
10	Awakening / REM	1.108	0.088	324	1.289	0.698

b) Pial artery, cardiac, Lumen

Amplitude

	contrast	ratio	SE	df	t.ratio	p.value
1	NREM / Quiet	1.051	0.040	472	1.328	0.674
2	IS / Quiet	1.094	0.041	472	2.386	0.121
3	IS / NREM	1.041	0.024	472	1.768	0.393
4	REM / Quiet	1.040	0.110	472	0.369	0.996
5	REM / NREM	0.989	0.100	472	-0.109	1.000
6	REM / IS	0.950	0.094	472	-0.513	0.986
7	Awakening / Quiet	1.019	0.085	472	0.229	0.999
8	Awakening / NREM	0.969	0.076	472	-0.394	0.995
9	Awakening / IS	0.931	0.074	472	-0.901	0.896
10	Awakening / REM	0.980	0.124	472	-0.159	1.000

Period

	contrast	ratio	SE	df	t.ratio	p.value
1	NREM / Quiet	1.037	0.017	468	2.258	0.161
2	IS / Quiet	1.057	0.017	468	3.414	0.006
3	IS / NREM	1.020	0.008	468	2.469	0.099
4	REM / Quiet	0.911	0.035	468	-2.419	0.112
5	REM / NREM	0.879	0.031	468	-3.614	0.003
6	REM / IS	0.861	0.030	468	-4.244	0.0003
7	Awakening / Quiet	0.955	0.041	468	-1.074	0.820
8	Awakening / NREM	0.922	0.039	468	-1.946	0.295
9	Awakening / IS	0.904	0.038	468	-2.420	0.112
10	Awakening / REM	1.049	0.055	468	0.908	0.894

Supplementary Figure 17

a) Penetrating arteriole, respiratory, Lumen

Amplitude

	contrast	ratio	SE	df	t.ratio	p.value
1	NREM / Quiet	1.144	0.395	50	0.389	0.995
2	IS / Quiet	1.227	0.401	50	0.627	0.970
3	IS / NREM	1.073	0.259	50	0.292	0.998
4	REM / Quiet	1.488	0.584	50	1.013	0.848
5	REM / NREM	1.301	0.445	50	0.770	0.938
6	REM / IS	1.212	0.409	50	0.571	0.979
7	Awakening / Quiet	1.956	1.006	50	1.304	0.690
8	Awakening / NREM	1.710	0.816	50	1.124	0.793
9	Awakening / IS	1.593	0.739	50	1.005	0.852
10	Awakening / REM	1.314	0.678	50	0.530	0.984

Period

	contrast	ratio	SE	df	t.ratio	p.value
1	NREM / Quiet	0.875	0.147	46	-0.797	0.930
2	IS / Quiet	0.903	0.145	46	-0.634	0.969
3	IS / NREM	1.032	0.126	46	0.261	0.999
4	REM / Quiet	0.918	0.188	46	-0.417	0.993
5	REM / NREM	1.049	0.185	46	0.270	0.999
6	REM / IS	1.016	0.170	46	0.095	1.000
7	Awakening / Quiet	0.904	0.230	46	-0.396	0.995
8	Awakening / NREM	1.033	0.243	46	0.138	1.000
9	Awakening / IS	1.001	0.229	46	0.003	1
10	Awakening / REM	0.985	0.258	46	-0.058	1.000

b) Penetrating arteriole, cardiac, Lumen

Amplitude

	contrast	ratio	SE	df	t.ratio	p.value
1	NREM / Quiet	1.300	0.197	120	1.729	0.420
2	IS / Quiet	1.188	0.171	120	1.194	0.755
3	IS / NREM	0.914	0.082	120	-0.999	0.856
4	REM / Quiet	0.935	0.189	120	-0.332	0.997
5	REM / NREM	0.720	0.124	120	-1.911	0.317
6	REM / IS	0.787	0.129	120	-1.460	0.590
7	Awakening / Quiet	1.284	0.280	120	1.146	0.782
8	Awakening / NREM	0.988	0.186	120	-0.066	1.000
9	Awakening / IS	1.081	0.196	120	0.429	0.993
10	Awakening / REM	1.373	0.316	120	1.377	0.644

Period

	contrast	ratio	SE	df	t.ratio	p.value
1	NREM / Quiet	0.948	0.049	124	-1.026	0.843
2	IS / Quiet	0.928	0.047	124	-1.460	0.590
3	IS / NREM	0.979	0.030	124	-0.679	0.961
4	REM / Quiet	0.765	0.048	124	-4.261	0.0004
5	REM / NREM	0.807	0.041	124	-4.234	0.0004
6	REM / IS	0.824	0.040	124	-3.945	0.001
7	Awakening / Quiet	0.781	0.053	124	-3.636	0.004
8	Awakening / NREM	0.824	0.056	124	-2.825	0.043
9	Awakening / IS	0.841	0.057	124	-2.568	0.083
10	Awakening / REM	1.021	0.079	124	0.267	0.999

Supplementary Figure 18

a) Penetrating arteriole, Lumen, Period

VLF

	contrast	ratio	SE	df	t.ratio	p.value
1	NREM / Quiet	1.097	0.037	290	2.710	0.055
2	IS / Quiet	1.077	0.037	290	2.159	0.199
3	IS / NREM	0.982	0.021	290	-0.859	0.912
4	REM / Quiet	1.201	0.044	290	5.021	0.00001
5	REM / NREM	1.096	0.027	290	3.739	0.002
6	REM / IS	1.116	0.028	290	4.433	0.0001
7	Awakening / Quiet	1.105	0.068	290	1.606	0.495
8	Awakening / NREM	1.007	0.057	290	0.128	1.000
9	Awakening / IS	1.026	0.059	290	0.442	0.992
10	Awakening / REM	0.919	0.054	290	-1.422	0.614

LF

	contrast	ratio	SE	df	t.ratio	p.value
1	NREM / Quiet	1.074	0.035	292	2.211	0.178
2	IS / Quiet	1.079	0.035	292	2.354	0.131
3	IS / NREM	1.005	0.016	292	0.318	0.998
4	REM / Quiet	0.939	0.038	292	-1.566	0.520
5	REM / NREM	0.874	0.025	292	-4.753	0.00003
6	REM / IS	0.870	0.024	292	-4.976	0.00001
7	Awakening / Quiet	0.920	0.032	292	-2.395	0.120
8	Awakening / NREM	0.857	0.033	292	-4.055	0.001
9	Awakening / IS	0.853	0.033	292	-4.137	0.0004
10	Awakening / REM	0.980	0.045	292	-0.440	0.992

b) Pial artery, Lumen, Period

VLF

	contrast	ratio	SE	df	t.ratio	p.value
1	NREM / Quiet	1.096	0.031	555	3.234	0.011
2	IS / Quiet	1.048	0.031	555	1.620	0.485
3	IS / NREM	0.957	0.017	555	-2.499	0.092
4	REM / Quiet	1.325	0.059	555	6.363	0
5	REM / NREM	1.209	0.045	555	5.100	0.00000
6	REM / IS	1.264	0.048	555	6.226	0
7	Awakening / Quiet	1.056	0.055	555	1.062	0.826
8	Awakening / NREM	0.964	0.050	555	-0.708	0.955
9	Awakening / IS	1.008	0.052	555	0.147	1.000
10	Awakening / REM	0.797	0.049	555	-3.694	0.002

LF

	contrast	ratio	SE	df	t.ratio	p.value
1	NREM / Quiet	1.150	0.023	564	6.963	0
2	IS / Quiet	1.131	0.023	564	6.008	0.00000
3	IS / NREM	0.983	0.012	564	-1.444	0.600
4	REM / Quiet	1.041	0.034	564	1.225	0.737
5	REM / NREM	0.905	0.025	564	-3.621	0.003
6	REM / IS	0.920	0.025	564	-3.011	0.023
7	Awakening / Quiet	0.961	0.035	564	-1.096	0.809
8	Awakening / NREM	0.835	0.031	564	-4.804	0.00002
9	Awakening / IS	0.850	0.032	564	-4.337	0.0002
10	Awakening / REM	0.923	0.041	564	-1.795	0.377
