# Supplementary material for: Sleep cycle-dependent vascular dynamics in male mice and the predicted effects on perivascular cerebrospinal fluid flow and solute transport
Source: Nat Commun. 2023 Feb 20;14:953. doi: 10.1038/s41467-023-36643-5 (PMC9941497; doi:10.1038/s41467-023-36643-5)
Supplement: Supplementary file 3 — Description of Additional Supplementary Files [file 41467_2023_36643_MOESM3_ESM.pdf]

Description of Additional Supplementary Files

File Name: Supplementary Movie 1
Description: Two-photon recording of a penetrating arteriole in layer II/III somatosensory cortex across a sleep cycle. Scale bar 20 μm.
