# Supplementary material for: Sleep cycle-dependent vascular dynamics in male mice and the predicted effects on perivascular cerebrospinal fluid flow and solute transport
Source: Nat Commun. 2023 Feb 20;14:953. doi: 10.1038/s41467-023-36643-5 (PMC9941497; doi:10.1038/s41467-023-36643-5)
Supplement: Supplementary file 5 — Reporting Summary [file 41467_2023_36643_MOESM5_ESM.pdf]

nature portfolio

Corresponding author(s): Laura Bojarskaite and Rune Enger

Last updated by author(s): Jan 29, 2023

Reporting Summary

Nature Portfolio wishes to improve the reproducibility of the work that we publish. This form provides structure for consistency and transparency in reporting. For further information on Nature Portfolio policies, see our Editorial Policies and the Editorial Policy Checklist.

Statistics

For all statistical analyses, confirm that the following items are present in the figure legend, table legend, main text, or Methods section.

n/a	Confirmed	
☐	☒	The exact sample size (*n*) for each experimental group/condition, given as a discrete number and unit of measurement
☐	☒	A statement on whether measurements were taken from distinct samples or whether the same sample was measured repeatedly
☐	☒	The statistical test(s) used AND whether they are one- or two-sided<br>*Only common tests should be described solely by name; describe more complex techniques in the Methods section.*
☐	☒	A description of all covariates tested
☐	☒	A description of any assumptions or corrections, such as tests of normality and adjustment for multiple comparisons
☐	☒	A full description of the statistical parameters including central tendency (e.g. means) or other basic estimates (e.g. regression coefficient) AND variation (e.g. standard deviation) or associated estimates of uncertainty (e.g. confidence intervals)
☐	☒	For null hypothesis testing, the test statistic (e.g. *F*, *t*, *r*) with confidence intervals, effect sizes, degrees of freedom and *P* value noted<br>*Give P values as exact values whenever suitable.*
☒	☐	For Bayesian analysis, information on the choice of priors and Markov chain Monte Carlo settings
☐	☒	For hierarchical and complex designs, identification of the appropriate level for tests and full reporting of outcomes
☐	☒	Estimates of effect sizes (e.g. Cohen's *d*, Pearson's *r*), indicating how they were calculated

Our web collection on statistics for biologists contains articles on many of the points above.

Software and code

Policy information about availability of computer code

Data collection	Two-photon microscopy data: ScanImage Premium 2021, Vidrio Technologies<br>Electrophysiology: Labview.
Data analysis	Data analyses of two-photon data: Custom MATLAB code (https://github.com/GliaLab/PVS-Sleep-Project), a development of https://github.com/GliaLab/Begonia (all code provided in the PVS-Sleep-project repository), DOI: https://doi.org/10.5281/zenodo.7540534. P-T analyses (python code): https://github.com/GliaLab/PVSflow, DOI: https://doi.org/10.5281/zenodo.7579913. Statistics: Statistical analyses were conducted in R (version 4.0.5). The linear mixed effect models were fitted using the glmmTMB package, residual plots were constructed by the DHARMa package (https://CRAN.R-project.org/package=DHARMa), and contrasts computed by the emmeans package (https://CRAN.R-project.org/package=emmeans). Modelling (pyhton code): https://github.com/AlexandraVallet/PVSflow, DOI: https://doi.org/10.5281/zenodo.7579913.

For manuscripts utilizing custom algorithms or software that are central to the research but not yet described in published literature, software must be made available to editors and reviewers. We strongly encourage code deposition in a community repository (e.g. GitHub). See the Nature Portfolio guidelines for submitting code & software for further information.

Data

Policy information about availability of data

All manuscripts must include a data availability statement. This statement should provide the following information, where applicable:

- Accession codes, unique identifiers, or web links for publicly available datasets
- A description of any restrictions on data availability
- For clinical datasets or third party data, please ensure that the statement adheres to our policy

Source data are provided with this paper. The raw data generated in this study have been deposited in the NIRD Research Data Archive under accession code 2022.00038 (https://doi.org/10.11582/2022.00038). A scripts for downloading all files can be found at https://github.com/GliaLab/PVS-Sleep-Project. The processed data and results from simulations are available at https://doi.org/10.5281/zenodo.7579700.

Human research participants

Policy information about studies involving human research participants and Sex and Gender in Research.

Reporting on sex and gender	N/A
Population characteristics	N/A
Recruitment	N/A
Ethics oversight	N/A

Note that full information on the approval of the study protocol must also be provided in the manuscript.

Field-specific reporting

Please select the one below that is the best fit for your research. If you are not sure, read the appropriate sections before making your selection.

[x] Life sciences [ ] Behavioural & social sciences [ ] Ecological, evolutionary & environmental sciences

For a reference copy of the document with all sections, see nature.com/documents/nr-reporting-summary-flat.pdf

Life sciences study design

All studies must disclose on these points even when the disclosure is negative.

Sample size	Sample sizes were chosen based on typical sample sizes in the literature and previous studies from the lab (1. Bojarskaite, L. and Bjørnstad, D.M. et al. Nat Commun 11, 3240 (2020), 2. Åbjørsbråten K.S and Syverstad Skaaraas eLife 11:e75055. (2022) 3. Enger et al., Cerebral Cortex, 27:1, 24–33, (2017)).
Data exclusions	Mice with surgical complications or mice that did not accommodate to head-fixation were excluded. Linescan recording with insufficient fluorescent signal quality were excluded. Because of the existence of a global rigid motion of the tissues, the displacements on both sides of a single structure (vessel or endfoot tube) should have a strong positive correlation. If not, this indicates that one edge of the structure was not well detected by our data processing tool. Unrealistic observations were therefore filtered out based on the correlation coefficient (0.8 for lumen and 0.7 for endfoot tube) between the position of both sides.
Replication	The numbers of mice studied are reported in every figure legend. The main display items demonstrate the response of each vessel, clearly showing the variable response on an individual observation
Randomization	No randomization was performed as there was only WT mice and no particular intervention.
Blinding	No blinding was performed as there was only WT mice and no particular intervention

Reporting for specific materials, systems and methods

We require information from authors about some types of materials, experimental systems and methods used in many studies. Here, indicate whether each material, system or method listed is relevant to your study. If you are not sure if a list item applies to your research, read the appropriate section before selecting a response.

Materials & experimental systems

n/a	Involved in the study	
☒	☐	Antibodies
☒	☐	Eukaryotic cell lines
☒	☐	Palaeontology and archaeology
☐	☒	Animals and other organisms
☒	☐	Clinical data
☒	☐	Dual use research of concern

Methods

n/a	Involved in the study	
☒	☐	ChIP-seq
☒	☐	Flow cytometry
☒	☐	MRI-based neuroimaging

Animals and other research organisms

Policy information about studies involving animals; ARRIVE guidelines recommended for reporting animal research, and Sex and Gender in Research

Laboratory animals	Male GLT1-eGFP reporter mice of 8–10 weeks.
Wild animals	No wild animals were used in the study.
Reporting on sex	Only male mice were used.
Field-collected samples	No field collected samples were used in the study.
Ethics oversight	All procedures were approved by the Norwegian Food Safety Authority (project number: 11983 and 22187).

Note that full information on the approval of the study protocol must also be provided in the manuscript.
